# Hsp70-Hsp40 Chaperone Complex Functions in Controlling Polarized Growth by Repressing Hsf1-Driven Heat Stress-Associated Transcription

**DOI:** 10.1371/journal.pgen.1003886

**Published:** 2013-10-17

**Authors:** Aleksandar Vjestica, Dan Zhang, Jianhua Liu, Snezhana Oliferenko

**Affiliations:** 1 Temasek Life Sciences Laboratory, Singapore; 2 Department of Biological Sciences, National University of Singapore, Singapore; 3 Genome Institute of Singapore, Singapore; The University of North Carolina at Chapel Hill, United States of America

## Abstract

How the molecular mechanisms of stress response are integrated at the cellular level remains obscure. Here we show that the cellular polarity machinery in the fission yeast *Schizosaccharomyces pombe* undergoes dynamic adaptation to thermal stress resulting in a period of decreased Cdc42 activity and altered, monopolar growth. Cells where the heat stress-associated transcription was genetically upregulated exhibit similar growth patterning in the absence of temperature insults. We identify the Ssa2-Mas5/Hsp70-Hsp40 chaperone complex as repressor of the heat shock transcription factor Hsf1. Cells lacking this chaperone activity constitutively activate the heat-stress-associated transcriptional program. Interestingly, they also exhibit intermittent monopolar growth within a physiological temperature range and are unable to adapt to heat stress. We propose that by negatively regulating the heat stress-associated transcription, the Ssa2-Mas5 chaperone system could optimize cellular growth under different temperature regiments.

## Author Summary

Heat stress, caused by fluctuations in ambient temperature, occurs frequently in nature. How organisms adapt and maintain regular patterns of growth over a range of environmental conditions remain poorly understood. Our work in the simple unicellular yeast *Schizosaccharomyces pombe* suggests that the heat stress-associated transcription must be repressed by the evolutionary conserved Hsp70-Hsp40 chaperone complex to allow cells to adapt the polarized growth machinery to elevated temperature.

## Introduction

Sensing ecological parameters and mounting an appropriate adaptive response allows organisms to thrive in changing habitats. Free-living microorganisms, such as yeast, can maintain regular patterns of growth over a relatively wide range of environmental temperatures. Cellular adaptation to increased temperature is one of the most robust and evolutionarily conserved mechanisms (reviewed in [1]). This response involves temperature-induced activation of genes encoding so-called heat shock proteins (HSPs) [1]–[4]. Molecular chaperones constitute a predominant class of HSPs and their up-regulation is essential for maintaining protein homeostasis at elevated temperatures (reviewed in [1]). Although much of heat shock-associated transcription is transient, recent analyses indicate deviation from this dynamic pattern and existence of genes with expression constitutively regulated by temperature [1], [5], [6].

One of the early events in the heat stress pathway ultimately setting off an extensive downstream gene expression program involves activation of critical transcriptional regulators, the heat shock factors (HSFs) [7], [8]. HSFs are evolutionary conserved winged helix-turn-helix proteins that bind to cis-acting DNA promoter sequences called heat shock elements (HSEs) [9]. HSFs are essential for viability in many fungi and control important developmental processes in higher eukaryotes suggesting that they may regulate basal transcription in addition to their function in stress response [10]–[14]. The ability of HSFs to respond to cellular stresses is under negative regulation by chaperones, modulation of nucleocytoplasmic shuttling, post-translational modifications and, in higher eukaryotes, transition from monomeric to trimeric state (reviewed in [15]). HSF repression by the Hsp90 and the Hsp70s-Hsp40 chaperone complexes [16]–[18] could provide a negative feedback loop titrating production of chaperones to allow for optimal protein folding [19]. Although essential for orchestrating an acute response to changing environment, constitutive Hsf1 transcriptional induction is known to retard cellular growth [20]. However, molecular mechanisms reducing HSF activity following the original heat-induced surge are poorly understood. Although fascinating, the cellular biology of adaptation to growth at elevated temperatures is largely unchartered.

**Figure 1:**
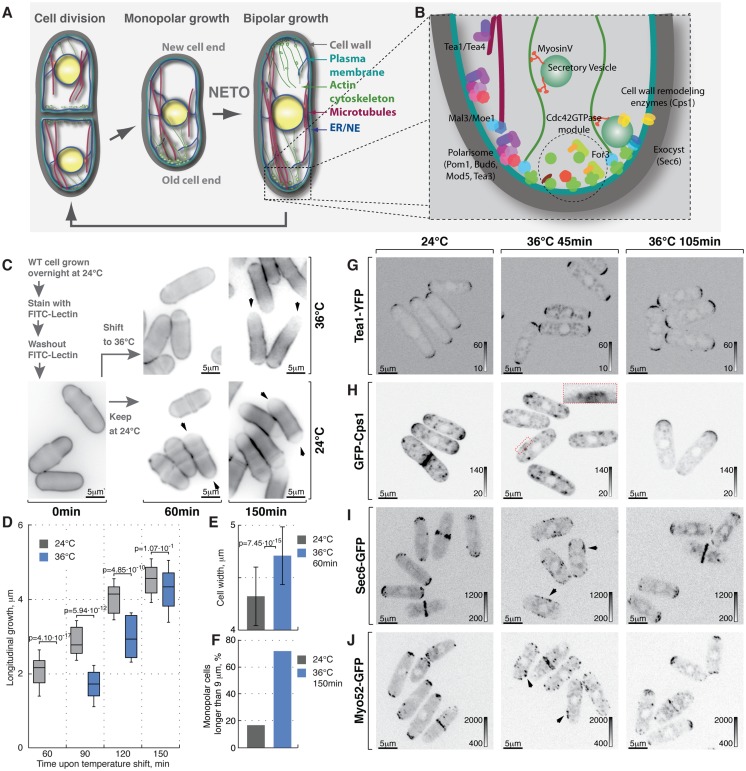
Heat-stress induces transient longitudinal growth arrest succeeded by monopolar growth. (**A**) Schematic of fission yeast cell cycle and (**B**) key regulators of cell growth and polarity. (**C**) Shown are single *z*-plane epifluorescence micrographs of FITC-Lectin stained cells grown at indicated temperatures and imaged immediately (left panel), 60 min (middle panels) and 150 min upon dye washout (right panels). (**D**) Quantification of longitudinal cell growth of cells represented in (**C**). Presented are box plots with standard deviation for cells grown at 24°C (gray) or shifted to 36°C (blue) for indicated time points; n>25 per sample per time point, p-values derived by Welch's test. (**E**) Cell width in cells grown at 24°C (gray) or shifted to 36°C for 60 min (blue). (**F**) Percentage of monopolar late G_2_ phase cells in a cell suspensions treated as described in (C), n>250 per sample. (**G–J**) Shown are whole cell maximum intensity *z*-projections of confocal micrographs of log-phase wild type cells expressing indicated fluorophore tagged marker proteins grown at 24°C (left panels) or shifted to 36°C for 45 min (middle panels) or 105 min (right panels). The arrows point out localization of the marker proteins to lateral cell cortex. Inset represents a zoom-in of the indicated region. Image contrast is reported using corresponding gray wedges. Scale bars, 5 µm.

The fission yeast *Schizosaccharomyces pombe* (*S. pombe*) are rod-shaped cells that grow at cell tips and divide by placing a medial septum. Upon division, each daughter cell initiates growth at the cell tip inherited from the mother, the so-called old-end (Fig. 1A). During the G_2_ phase of the cell cycle, cells transition to bipolar growth in a process termed New-End-Take-Off (NETO) [21]. NETO occurs when growth sets a sufficient distance between the cell tips [21], [22] and requires the function of a large protein complex called polarisome and active actin cytoskeleton remodeling [23]–[25]. The polarisome, built around the kelch-repeat protein Tea1 and its binding partner Tea4, ensures the fidelity of polarized growth through recruitment of downstream polarity factors (Fig. 1B and [23], [24], [26], [27]).

Polarized growth requires local plasma membrane (PM) and cell wall remodeling. The targeted secretion of cell wall enzymes, such as the β-glucan synthase Cps1 [28] relies on long-range exocytic vesicle transport along actin cables and the tethering of the secretory vesicles to the PM by the exocyst complex [29]. Polarized assembly of both actin cables and the exocyst depends on the Rho family GTPase Cdc42 [30]–[34].

The guanine-nucleotide-exchange-factors (GEFs) promote Cdc42 activity while the GTPase-activating proteins (GAPs) repress it [35], [36]. The fission yeast GEFs, Scd1 and Gef1, are enriched at the cell tips [37], [38], while the Cdc42 GAP Rga4 localizes to the lateral cortex [39]. This results in predominant activation of Cdc42 at the cell poles. Recent high-end imaging techniques have uncovered oscillations in Cdc42 activity at the PM suggesting that spatiotemporal activation of Cdc42 is governed by a series of positive and negative feedback loops [40]–[43]. The polarisome and the Cdc42 circuitries appear to intersect at various levels [26], [39], [44].

The mechanisms regulating polarized growth exhibit a high degree of plasticity [45], [46]. Yeast cells readily orient growth towards their mating partners [47], [48] and relocalize the growth machinery to the site of cell injury [49]. In *Saccharomyces cerevisiae* (*S. cerevisiae*), heat stress activates the cell wall integrity pathway and promotes nucleotide exchange of the Rho-family GTPase Rho1, which in turn initiates F-actin dependent redistribution of β-glucan synthase throughout the entire cellular cortex [50], [51]. Repolarization of actin as cells adapt to elevated temperature appears to depend on a negative feedback mechanism where the protein kinase C Pkc1 activates MAPK cascade to inhibit the Rho1-GEF [52]. In fission yeast several types of stress, including heat and osmolytes, also cause F-actin redistribution throughout the cellular cortex [53]–[56] and lead to activation of MAP kinases [57]–[59].

Here we utilize a multi-disciplinary approach to explore how the heat stress and associated transcription impact polarized growth in fission yeast. We identify the Mas5-Ssa2 chaperone complex as a negative regulator of the heat shock transcription factor Hsf1. Using cells lacking the chaperone function we probe the physiological outcome of constitutively upregulated heat stress response.

## Results

### Heat stress triggers transient loss of polarity followed by a period of monopolar growth in fission yeast cells exposed to elevated temperature

Lectins bind polysaccharides of the yeast cell wall with high affinity but without impairing cell growth [60]. Thus, sites of cell wall deposition subsequent to fluorophore-coupled lectin staining can be readily visualized as a dip in fluorescence intensity [61]. To monitor cell growth upon heat stress we stained log-phase wild type cells growing at 24°C with FITC-Lectin, washed-out the excess dye and immediately shifted cells to 36°C or allowed them to grow at 24°C (Fig. 1C). Samples were collected at 30-minute intervals, fixed and subjected to epifluorescence microscopy. Cells continuously growing at 24°C elongated at a rate of 2.51±0.77 mm per hour after the dye washout. Conversely, cells shifted to 36°C for one hour fully arrested longitudinal growth (Fig. 1C and 1D, n>60 cells per sample, p^Welch's t-test^ = 4.10⋅10^−17^). Interestingly the diameter of heat-stressed cells increased (Fig. 1C and 1E, width increased by 9±7%, n>60 cells per sample, p^Welch's t-test^ = 7.45⋅10^−15^) and the overall FITC-Lectin staining of the cell visibly decreased (data not shown) suggesting that the cell wall underwent remodeling throughout the cell periphery rather than at the cell tips.

After a period of depolarized cell wall deposition, cells resumed polarized growth and subsequently elongated at a rate of ∼28% greater than cells grown at 24°C (Fig. 1D). Most control cells in the G_2_ phase of the cell cycle exhibited bipolar growth pattern (Fig. 1C and 1F). In line with previous observations [21], the majority of heat-stressed cells elongated only at one cell tip (Fig. 1C and 1F). When propagated overnight at 36°C, most wild type cells became bipolar (83%; Fig. S1A). Thus, the heat stress leads to a transient arrest of longitudinal growth followed by regaining cellular polarity through an intermediate monopolar growth phase.

Temperature had no effect on the distribution of the polarisome components Tea1-YFP, Tea4-GFP and Pom1-GFP as these markers exhibited bipolar localization in late G_2_ phase cells at both 24°C and 36°C within the observed timeframe (Fig. 1G, S1B, S1C, see Fig. S1F for quantifications). In contrast, β-glucan synthase GFP-Cps1, which predominantly localized to both cell tips in late G_2_ cells at 24°C, could be observed in intracellular vesicle-like structures and at the lateral cell cortex 45 minutes after cells were shifted to 36°C (Fig. 1H). Longer incubation at 36°C (105 minutes) allowed all cells to repolarize GFP-Cps1 to the cell tips, albeit in a monopolar fashion (Fig. 1H and S1F). Similarly, the exocyst component Sec6-GFP localized to both cell tips in unstressed late G_2_ phase cells but was observed throughout the cell cortex in cells shifted to 36°C for 45 minutes (Fig. 1I). Cells growing at 36°C for 105 minutes did repolarize Sec6-GFP but approximately half of late G_2_ phase cells confined it to only one tip (Fig. 1I and S1F).

Polarization of actin structures typical for yeast cells grown at normal temperatures was completely abolished following incubation at 36°C for 45 minutes. Cells were able to re-polarize F-actin cytoskeleton after 105 minutes at 36°C but it was unequally distributed between the two tips of most late G_2_ phase cells (Fig. S1D, S1F and S1G). Similarly, both fluorophore-tagged formin For3 and the type-V myosin Myo52 relocalized from the tips to the entire cortex upon heat stress but then re-established polarity after 105 minutes at 36°C, although in a monopolar fashion (Fig. 1J, S1E and S1F).

Taken together our results suggest that processes confining the growth machinery to the polarisome-demarcated cell tips are rapidly perturbed by temperature increase. Cells become monopolar in the adaptive phase following the heat stress but eventually resume normal bipolar growth.

### Cortical association of GTP-bound Cdc42 and its GEFs and GAP is modulated by temperature

**Figure 2:**
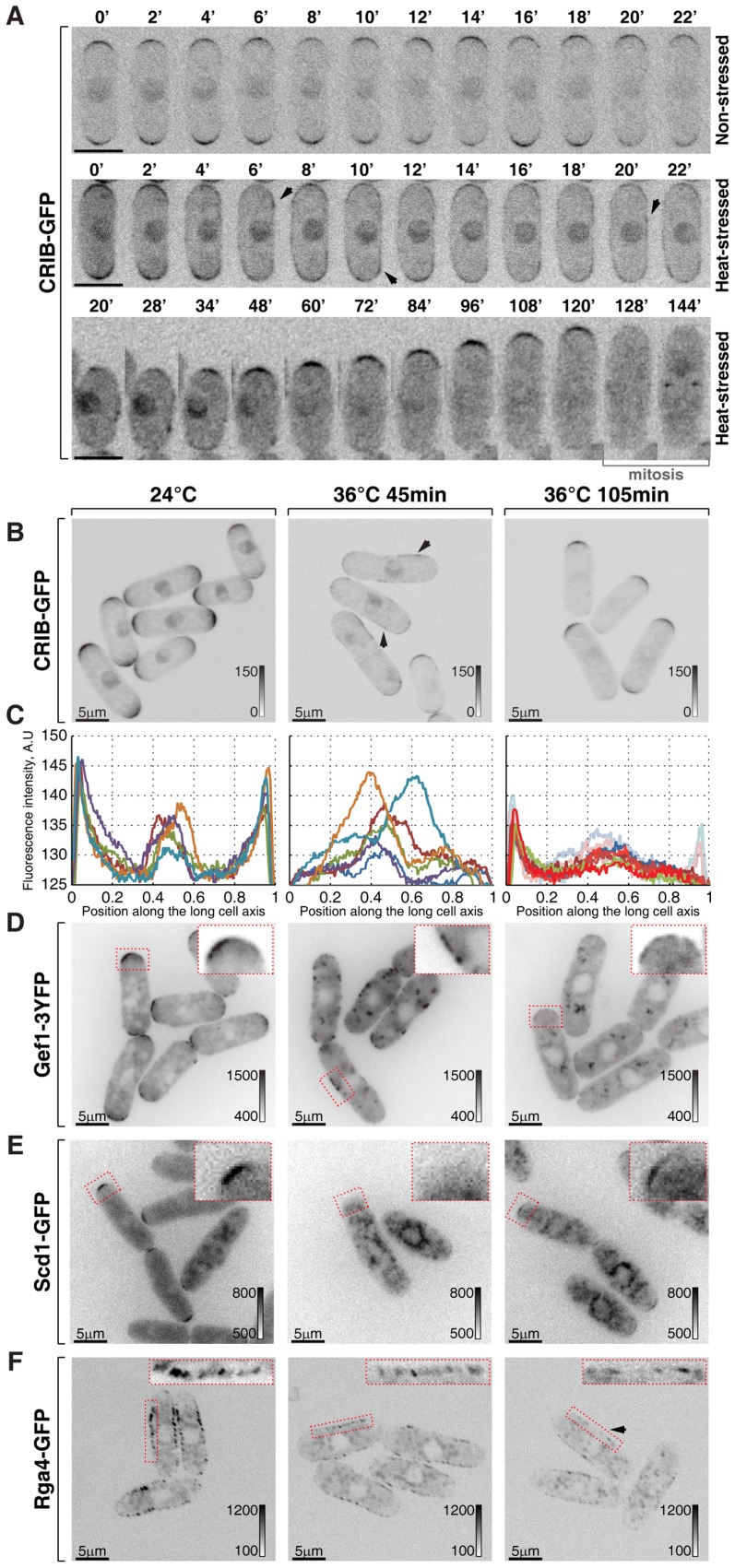
Cortical association of GTP-bound Cdc42 and its GEFs and GAP is modulated by temperature. (**A**) Single *z*-plane spinning disk confocal microscopy time-lapse images of CRIB-GFP expressing wild type cells grown at room temperature (top panel) or shifted to 36°C for the indicated period of time (middle and bottom panels). Spreading of CRIB-GFP signal immediately following heat stress (middle panel) is indicated by arrowheads. A heat-stressed cell shown in the bottom panel repolarizes CRIB-GFP to one cell tip and eventually enters mitosis. (**B**) Shown are whole cell maximum intensity *z*-projections of scanning confocal micrographs of log-phase wild type cells expressing CRIB-GFP grown at 24°C (left panels) or shifted to 36°C for 45 min (middle panels) or 105 min (right panels). The arrows point out localization of the marker protein to lateral cell cortex. (**C**) Quantification of CRIB-GFP intensities along the long cell axis in log-phase wild type cells grown under indicated temperature regiments. Individual lines correspond to individual cells. Low opacity lines refer to heat-stressed cells from the subpopulation exhibiting bipolar CRIB-GFP localization after 105 min at 36°C. (**D–F**) Shown are single *z*-plane micrographs of log-phase wild type cells expressing indicated fluorophore-tagged Cdc42 regulators grown at 24°C (left panels) or shifted to 36°C for 45 min (middle panels) or 105 min (right panels). The insets represent zoomed-in and re-contrasted main image segments outlined in red dotted line. Image contrast is reported using corresponding gray wedges. Scale bars, 5 µm.

Both F-actin distribution and exocyst localization are governed by the activity of a Rho-family GTPase Cdc42 [29]. To monitor the active Cdc42 *in vivo* during heat-stress we used the GFP-tagged CRIB (Cdc42/Rac-interactive binding) domain reported to interact with the GTP-bound Cdc42 [39]. G_2_ phase cells grown at 24°C focus the Cdc42 activity to the cell tips with moderate oscillations in its levels ([40] and Fig. 2A, top panel). Minutes after cells were shifted from 24°C to 36°C, CRIB-GFP was seen spreading from the cell tips towards the cell equator establishing zones of Cdc42-activity at the cell sides (Fig. 2A, middle panel). After a period of depolarization, cells restricted CRIB-GFP to just one tip and initiated monopolar growth (Fig. 2A, bottom panel). In contrast to the bipolar localization in almost all of G_2_ phase cells grown at 24°C, a third of G_2_ phase cells grown at 36°C for 105 minutes exhibited a strictly monopolar CRIB-GFP pattern (Fig. 2B and S2A). While total fluorescence signal of CRIB-GFP was not perturbed by temperature, its cortical association was decreased as compared to unstressed cells (Fig. 2C).

Both Cdc42-GEFs exhibited a bipolar localization pattern in unstressed late G_2_ cells (Fig. 2D, 2E and S2A). Forty-five minutes upon temperature shift from 24°C to 36°C, Gef1-3YFP localized to patches distributed throughout the plasma membrane whereas Scd1-GFP was no longer detectable at the cell cortex (Fig. 2D and 2E). Both Gef1-3YFP and Scd1-GFP also localized to intracellular structures upon heat stress. After 105 minutes at 36°C, both GEFs became confined to cell tips but in a monopolar fashion (Fig. 2D, 2E and S2A). Interestingly, Gef1-3YFP and Scd1-GFP levels were severely diminished at the cortex of cells repolarizing growth at elevated temperature. The average intensity of Scd1-GFP at a single cell tip decreased by 49±23% (p^Welch's t-test^ = 1.43⋅10^−7^, n>25 cells per sample), whereas Gef1-3YFP signal dipped by 65±17% (p^Welch's t-test^ = 7.34⋅10^−12^, n>25 cells per sample).

The only known fission yeast Cdc42-GAP, Rga4, localizes to the lateral cell cortex and to the new cell end in pre-NETO cells [39]. Upon heat stress, the Rga4-GFP localization remained restricted to the cell sides throughout the course of the experiment although its absolute amount at the cortex progressively decreased (Fig. 2F; after 105 minutes at 36°C the cortex-associated Rga4-GFP signal fell by 34±22%, p^Welch's t-test^ = 7.35⋅10^−8^, n>25 cells per sample). The diminished cortical levels of Cdc42 regulators in cells shifted to the elevated temperature were not a consequence of a gross decrease in total amounts of these proteins (Fig. S2B).

Since heat-stressed fission yeast cells eventually resume bipolar growth (Fig. S1A), we analyzed cortical levels of Cdc42-GAP and GEFs in cells growing overnight at 36°C. Gef1-3YFP and Scd1-GFP did assume bipolar localization in late G_2_ cells (Fig. S2E–F). Surprisingly, the average signal intensity per cell tip remained relatively low, as did Rga4-GFP levels at the lateral cortex. In fact, even though levels of active Cdc42 at the cortex were similar in cells growing at 18°C, 24°C and 30°C (Fig. S2C and S2D), the cortical levels of the three Cdc42 regulators inversely correlated with the growth temperature (Fig. S2E–G). Immunoblot analyses of cells grown to log-phase at 18°C, 24°C, 30°C and 36°C showed a similar trend in total protein levels of Scd1-GFP, Gef1-3YFP and Rga4-GFP (Fig. S2H).

Thus, cortical Cdc42 activity appears to be extensively regulated at both initial and adaptive phases of the heat-stress response and during steady state growth at different temperatures.

### Overexpression of the heat shock transcription factor Hsf1 leads to monopolar growth

**Figure 3:**
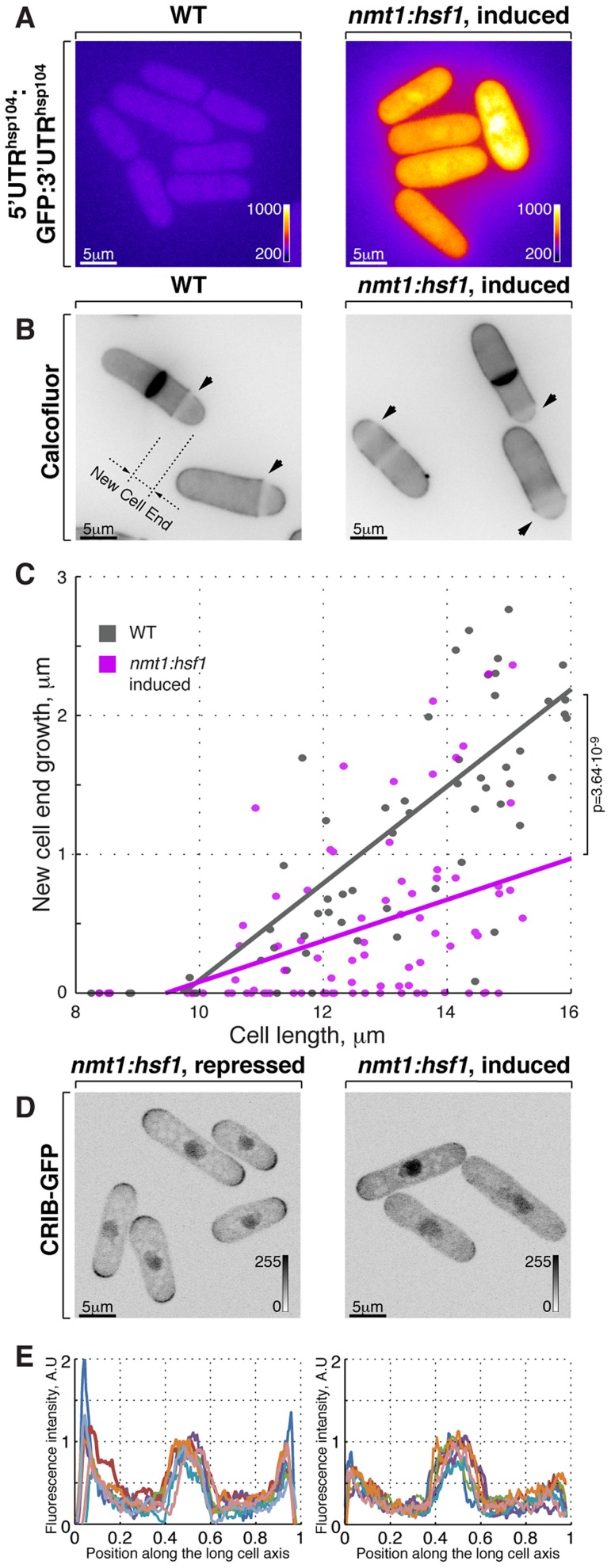
Hsf1-mediated transcription regulates growth patterning in fission yeast. (**A**) GFP reporter expression is driven by *hsp104* promoter in cells where Hsf1 ORF is under the control of either wild type or *nmt1* promoter grown to log phase in minimal medium without thiamine. Shown are pseudocolored average intensity whole cell z-projections of epifluorescence image stacks. Image contrast is reported using corresponding color wedges. (**B**) Single z-plane images of calcofluor stained wild type and cells with Hsf1 over-expression driven by *nmt1* promoter induced for 20 hours by removal of thiamine. Arrowheads point to birth-scars; the new-cell-end length was determined as a distance from the birth-scar to the proximal cell tip. (**C**) New-cell-end length as a function of cell length for wild type (gray) and cells over-expressing Hsf1 from the thiamine responsive *nmt1* promoter (magenta). Dots represent individual late G_2_ phase cells and lines represent linear regressions for cells analyzed (n>30). p-values were obtained through ANCOVA analysis. (**D**) Shown are whole cell maximum intensity *z*-projections of scanning confocal micrographs of *nmt1::hsf1* cells expressing CRIB-GFP and grown in the presence (left panel) or absence (right panel) of thiamine. (**E**) Quantification of CRIB-GFP intensities along the long cell axis in log-phase cells grown under conditions described in (**D**). Gray wedges report image contrasting. Scale bars, 5 µm.

Cells respond to stress by adjusting their transcriptional output [1], [62]. Heat-stress associated transcription is primarily mediated by a temporary activation of the HSF family of transcription factors (reviewed in [63]), Hsf1 is the only HSF homologue present in the fission yeast genome and it was reported to be essential not only for heat shock response but also for vegetative growth [11], [64]. We confirmed that cells depleted of Hsf1 ceased growth (Fig. S3A). We wondered if cell polarity could be affected by the Hsf1 transcriptional activity alone, without the temperature change. To this aim, we replaced the fission yeast *hsf1* genomic promoter with a strong, thiamine responsive *nmt1* promoter. Cells with high levels of Hsf1 eventually arrested growth (Fig. S3B). Overexpression of Hsf1 was sufficient to promote its transcriptional activity since we observed vastly increased levels of the heat stress-induced protein Hsp104 (Fig. S3C–D) and the GFP reporter expressed under *hsp104* regulatory elements (Fig. 3A and S3E, see Materials and Methods for details on reporter construction). Strikingly, Calcofluor staining revealed that Hsf1-overexpressing cells became increasingly monopolar (Fig. 3B and 3B; on average the new-end growth decreased by 59±22% in *nmt1:hsf1* cells as compared to wild type cells, p^Welch's t-test^ = 4.58⋅10^−10^, n>50 cells per sample). Furthermore, cells overexpressing Hsf1 exhibited decreased tip association of CRIB-GFP that predominantly localized to only one cell tip (Fig. 3D and 3E). Taken together, it appears that high Hsf1 activity prevents normal growth patterning and eventually leads to growth arrest in fission yeast.

### The Ssa2-Mas5 chaperone complex modulates adaptation to heat stress in fission yeast

**Figure 4:**
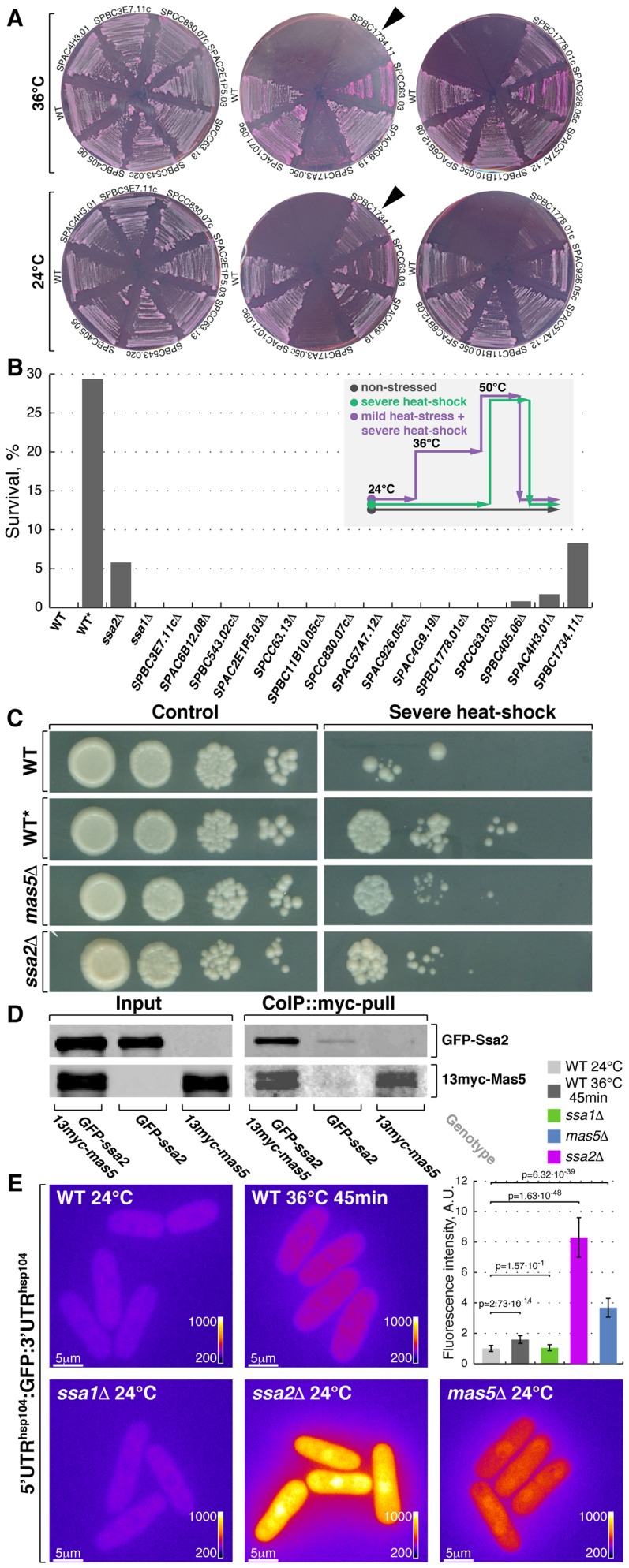
Screening for chaperone mutant cells with elevated levels of heat stress-associated transcription. (**A**) Fission yeast strains lacking indicated non-essential nucleocytoplasmic Hsp40 proteins were streaked out onto YES plates containing phloxine B and grown at 24°C or 36°C. Arrowheads points to the strain lacking DnaJ chaperone Mas5. (**B**) Survival rate upon extreme heat-shock (50°C for 15 min) of wild type cells (WT), wild type cells pre-exposed to mild heat stress (WT^*^) and cells lacking individual SSA subfamily chaperones or indicated non-essential nucleocytoplasmic Hsp40 proteins. The inset outlines the screening strategy used to identify strains with elevated levels of heat-stress associated transcription: cells were grown to log-phase at 24°C and aliquots were either allowed to continue growth at 24°C (gray line) or shifted to 50°C for 15 min (green line). An additional sample of wild type cells was shifted to 36°C for 45 min before being transferred to 50°C for 15 min (purple line). Number of colony forming units was measured for each sample and used to calculate survival rates. (**C**) Dilution spotting assay of wild type cells (WT), wild type cells pre-exposed to mild heat-stress (WT^*^) and cells lacking Mas5 or Ssa2 grown at 24°C (left panels) and exposed to 50°C for 15 min prior to plating (right panels). (**D**) Lysates prepared from cells with indicated genotypes were incubated with *anti*-myc antibodies and subsequently with beads coupled to Protein-G. Proteins that remained associated with the beads after multiple buffer washes were resolved by SDS-PAGE and prepared for Western Blotting with *anti*-myc and *anti*-GFP antibodies. (**E**) Pseudocolored average *z*-projection epifluorescence images of the GFP under the regulation of *hsp104* regulatory elements in wild type, *ssa1Δ*, *ssa2Δ* and *mas5Δ* cells grown in indicated conditions. Image contrast is reported using corresponding color wedges. Scale bars, 5 µm. Top right panel histogram quantifies the fluorescence signal in indicated strains.

Hsp90 and Hsp70-Hsp40 chaperones are known repressors of Hsf1 activity but both Hsp90 and Hsp70 proteins are known to engage a large number of client proteins [65]. To isolate specificity factor(s) for Hsp70 that could function in this process, we screened the deletion library of sixteen non-essential genes encoding nucleocytoplasmic Hsp40 chaperones (Supplementary Table S2). Two screening strategies were employed. One approach was based on our finding that Hsf1 overexpression leads to decreased growth rates and eventually a growth arrest. We hypothesized that mutant strains with elevated levels of Hsf1-associated transcription would display decreased growth rates already at 24°C and would not be able to grow at 36°C due to high Hsf1 activity. Within the deletion library of nonessential Hsp70-binding proteins only the strain lacking the DnaJ chaperone Mas5 exhibited such growth pattern (Fig. 4A).

The second screening strategy exploited the fact that a mild heat stress increases survival of cells exposed to a subsequent severe transient heat shock, likely due to higher expression of protective factors in pre-stressed cells [66]. As outlined in Fig. 4B, wild type cells and deletion mutants were grown to log-phase at 24°C and culture aliquots were either allowed to continue growth at 24°C or shifted to 50°C for 15 minutes before returning them to 24°C. Additionally, a sample of wild type cells was shifted to 36°C for 45 minutes before being exposed to 50°C. Survival was measured by assessing the number of colony forming units in each culture (Fig. 4B) and confirmed by a dilution spotting assay (Fig. 4C). Wild type and most tested mutant strains exhibited a survival rate below 0.5% when shifted from 24°C to 50°C for 15 minutes (n>200 CFU per strain). Conversely, 29% of wild type cells survived if a mild heat stress preceded the severe heat shock. Interestingly, cells lacking Mas5 showed an 8% survival rate when shifted directly from 24°C to 50°C for 15 minutes (Fig. 4B and 4C).

Both major nucleocytoplasmic Hsp70 chaperones, Ssa1 and Ssa2, physically interacted with Mas5 as shown by co-immunoprecipitation experiments (Fig. 4D and S4A). Cells lacking Ssa2 but not Ssa1 exhibited increased survival following a transient heat shock, similar to *mas5Δ* cells (Fig. 4B and 4C). In line with this, the fluorophore-tagged marker proteins Hsp104, Swo1 and Psi1 that are known to be strongly upregulated upon heat stress were highly abundant in *mas5Δ* and *ssa2Δ* but not in *ssa1Δ* cells, again suggesting that Ssa2-Mas5 complex may have a specific function in heat stress response (Fig. S4B and data not shown). Excluding the possibility that elevated fluorescence levels were due to selective stabilization of heat-responsive proteins in mutant cells, the UTRs^Hsp104^:GFP reporter was also strongly elevated in both *ssa2Δ* and *mas5Δ* but not in *ssa1Δ* cells (Fig. 4E). Thus, *mas5Δ* and *ssa2Δ* cells express high levels of heat stress markers already at 24°C, which may increase their chances of survival following transient heat shock.

### Cells lacking the Mas5-Ssa2 chaperone complex exhibit an intermittent, monopolar growth pattern

**Figure 5:**
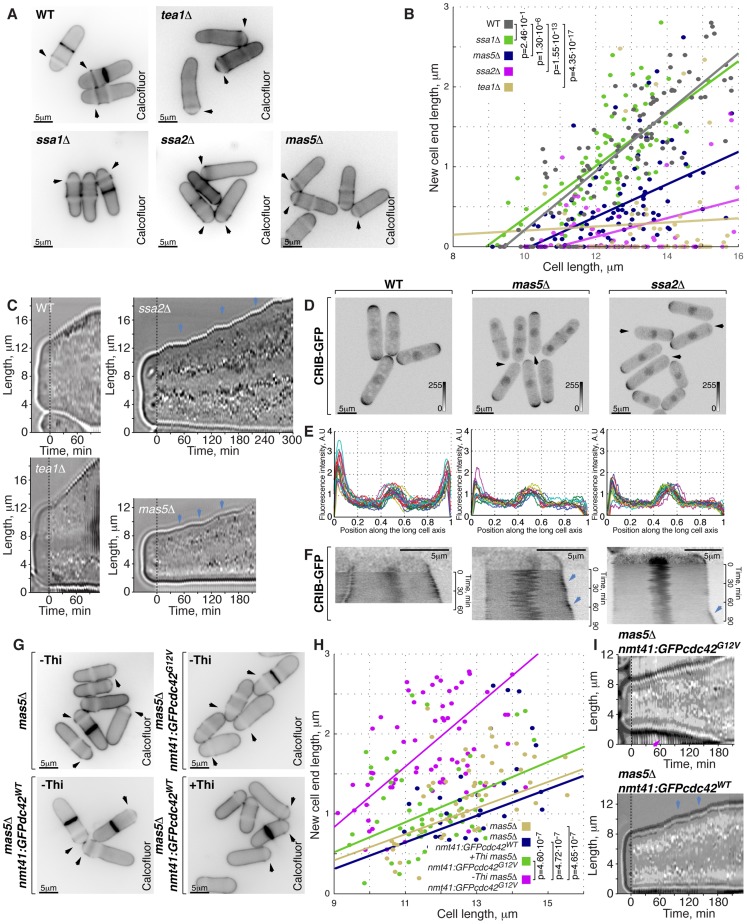
Cells with impaired Ssa2p•Mas5 complex have decreased cortical Cdc42 activity and exhibit monopolar intermittent growth pattern. (**A**) Single z-plane images of calcofluor stained log-phase cells with indicated genotypes. Arrows point to birth-scars. (**B**) New-cell-end length as a function of cell length for cells with indicated genotypes. Points represent individual cells, lines represent the linear regression of a sample and p-values were obtained through ANCOVA. (**C**) Kymographs of bright-field microscopy time-lapse analyses of cells with the indicated genetic backgrounds grown in a perfusion chamber at room temperature. Arrowheads point to growth pauses characteristic to *ssa2Δ* and *mas5Δ* cells. (**D**) Shown are whole cell maximum intensity *z*-projections of scanning confocal micrographs of log-phase cells with indicated genotypes expressing CRIB-GFP, arrowheads point to cell tips lacking Cdc42 activity. (**E**) Quantification of CRIB-GFP intensities along the long cell axis in log-phase wild type (left panel), *mas5Δ* (middle panel) and *ssa2Δ* cells (right panels). Individual lines correspond to individual cells. (**F**) Kymographs of single *z*-plane spinning disk confocal microscopy time-lapse of wild type (left panel), *mas5Δ* (middle panel) and *ssa2Δ* cells (right panels) grown at room temperature. Arrows point to intermittent Cdc42 activity bursts associated with longitudinal growth seen in *mas5Δ* and *ssa2Δ* cells. (**G**) Single z-plane images of calcofluor stained log-phase cells with indicated genotypes grown in minimal medium in the presence or absence of thiamine for 30 hours and shifted to YE medium for 7 hours prior to fixation and staining. Arrows point to birth-scars. (**H**) New-cell-end length as a function of total cell length for cells presented in (**G**). Points represent individual cells, lines represent the linear regression of a sample and p-values were obtained through ANCOVA. (**I**) Kymographs of bright-field microscopy time-lapse analyses of cells with the indicated genetic backgrounds grown in a perfusion chamber at room temperature. Blue arrowheads point to growth pauses and a magenta arrowhead indicates onset of bipolar growth. Scale bars, 5 µm.

Doubling time of log-phase *mas5Δ* and *ssa2Δ* cultures growing at 24°C increased approximately 1.3-fold and 2-fold as compared to the wild type (Fig. S5A left panel, p^Welch's t-test^ = 1.61⋅10^−2^ and 7.16⋅10^−3^, n = 3). Moreover, cells lacking Mas5 and Ssa2 divided at a consistently shorter length as compared to the wild type (Fig. S5A, right panel). Calcofluor staining suggested that both *ssa2Δ* and *mas5Δ* cells exhibited significantly decreased new-cell-end growth (Fig. 5A and 5B, on average, *mas5Δ* and *ssa2Δ* cells reduced the new-end growth by 50±24% and 78±93% respectively, p^Welch's t-test^ = 2.28⋅10^−28^ and 2.46⋅10^−11^, n>60 per genotype).

Progression through the cell cycle was not affected in *mas5Δ* and *ssa2Δ* mutants, as evaluated by FACS (Fluorescence Activated Cell Sorting) analysis (Fig. S5B), suggesting that the monopolar growth in these mutants could not be explained by delaying entry into the G_2_ phase of the cell cycle.

Subcellular distribution of the polarisome components Tea1-YFP, Tea4-GFP and Pom1-GFP was unaffected by *mas5* gene deletion (Fig. S5C–S5F). In contrast, localization of the β-glucan synthase GFP-Cps1 was predominantly monopolar late into the G_2_ phase of the cell cycle (Fig. S5C and S5G). Similarly, exocyst subunit Sec6 localized to only one cell tip in a majority of *mas5Δ* cells (Fig. S5G and S5K). Imaging of LifeAct-GFP [67] showed that the actin cytoskeletal structures were also enriched at just one cell tip of late G_2_ phase *mas5Δ* cells (Fig. S5I, S5C, S5J). Consistently, For3-GFP and Myo52-GFP were also monopolar in most *mas5Δ* cells longer than 9 µm (Fig. S5C, S5K and S5L).

Next, we performed live imaging of wild type, *tea1Δ*, *ssa2Δ* and *mas5Δ* cells grown in a perfusion chamber at room temperature (Supplemental Movie 1, see Materials and Methods). After an initial phase of monopolar growth, the newly born wild type cells underwent NETO and assumed bipolar growth pattern clearly seen in the kymograph (Fig. 5C). As expected [27] *tea1Δ* cells maintained a constitutively monopolar growth pattern and were rarely seen undergoing NETO (Fig. 5C). Consistent with the data presented earlier, over 90% of *ssa2Δ* and *mas5Δ* cells grew only at the old cell tip. Surprisingly, all mutant cells exhibited an intermittent growth pattern (Fig. 5C, n>10 cells) with periods of complete growth arrest lasting from 5 to over 30 minutes followed by growth bursts of 5 to over 30 minutes.

Active Cdc42, as visualized by CRIB-GFP, also localized to one cell tip in cells lacking Mas5 or Ssa2 (Fig. 5D, 3% of wild type, 93% of *ssa2Δ* and 58% of *mas5Δ* late G_2_ cells were monopolar). Furthermore, the absolute levels of CRIB-GFP signal at the polar cortex were diminished in *ssa2Δ* and *mas5Δ* cells (Fig. 5E). The time-lapse spinning-disk confocal microscopy revealed that the CRIB-GFP recruitment to the cell tips was unstable in *mas5Δ* and *ssa2Δ* cells (Fig. 5F). In wild type cells, CRIB-GFP signal at the continuously growing old cell end displayed only minor intensity oscillations during the course of imaging. On the other hand, CRIB-GFP levels at the new cell end underwent major on-off oscillations prior to NETO but eventually stabilized exhibiting only minor oscillations (Fig. 5F, left panel, also see [40]). However, CRIB-GFP levels underwent on-off oscillations at both tips throughout the cell cycle in *mas5Δ* and *ssa2Δ* cells. We observed that high CRIB-GFP levels at the old cell end coincided with periods of tip growth (Fig. 5F, middle and right panels). CRIB-GFP levels at the new cell end of *ssa2Δ* and *mas5Δ* cells underwent on-off oscillations that would rarely stabilize to allow for noticeable tip elongation during the course of imaging. These results suggest that the intermittent growth pattern of *ssa2Δ* and *mas5Δ* cells is a consequence of the unstable Cdc42 activity at the cell tips. Furthermore, the decreased Cdc42 activity at the new cell end could explain the monopolar growth pattern of mutants lacking Ssa2-Mas5 chaperone complex.

The low levels of active Cdc42 in *mas5Δ* cells prompted us to explore the distribution of the Cdc42-GAP and GEFs in the mutant. Both Gef1-3YFP and Scd1-GFP exhibited bipolar localization in majority of late wild type cells but were predominantly monopolar in cells lacking Mas5 (Fig. S5M, S5N and S5C). The Cdc42-GAP, Rga4-GFP, remained restricted to the lateral cell cortex in ∼90% in *mas5Δ* cells (Fig. S5O). Levels of cortically associated Cdc42 regulators appeared decreased in *mas5Δ* cells as compared to the wild type. The average intensity of Gef1-3YFP at a cell tip was decreased by 38±28% whereas Scd1-GFP signal diminished by 53±27% (p^Welch's t-test^ = 2.51⋅10^−6^ and 7.94⋅10^−7^ respectively, n>25 cells per sample). A 20±22% decrease in cortically associated levels was also observed for the Rga4-GFP in cells lacking Mas5 (p^Welch's t-test^ = 2.71⋅10^−4^, n>25 cells per sample). The total protein levels of the three examined Cdc42 regulators were also diminished in *mas5Δ* cells (Fig. S5P). Gef1-3YFP and Scd1-GFP levels decreased by 18% and 48% respectively and a 21% decrease was observed for Rga4-GFP.

These results suggested that the lack of Ssa2-Mas5 chaperone complex resulted in monopolar growth due to insufficient function of the Cdc42 GTPase module. Indeed, mild overexpression of the constitutively active Cdc42-G12V mutant but not wild type Cdc42 in *mas5Δ* cells led to its enrichment at the cell tips (Fig. S5Q) and a significant increase in bipolar growth (Fig. 5G and 5H, p = 4.65⋅10^−7^). Interestingly, upregulation of Cdc42 activity also largely rescued the intermittent growth phenotype of *mas5Δ* cells, as observed by time-lapse microscopy (Fig. 5I, continuous growth was sustained in 11/20 cells overexpressing Cdc42-G12V and only 3/20 cells overexpressing the wild type Cdc42).

We wondered if the abnormal monopolar growth observed in cells lacking Mas5-Ssa2 chaperone complex could be a manifestation of an upregulated heat stress response.

### Cells lacking Mas5-Ssa2 chaperone complex exhibit elevated levels of heat stress-associated transcription

**Figure 6:**
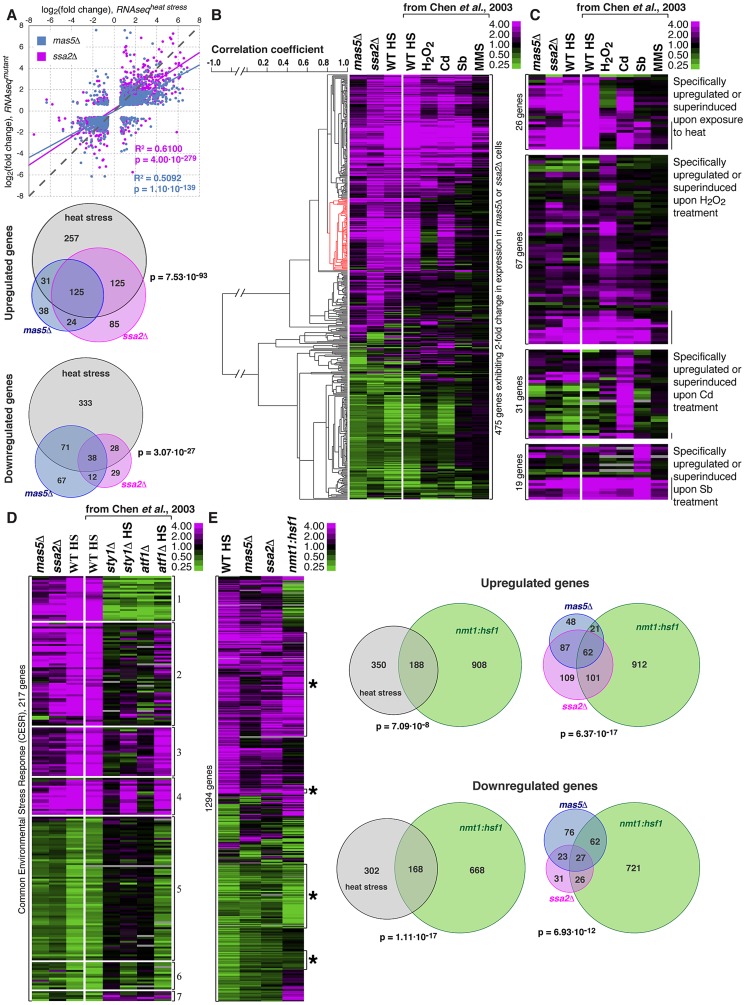
Cells with impaired function of the Ssa2p•Mas5 chaperone complex exhibit elevated levels of heat-stress associated transcription. (**A**) Top panel represents correlation analysis between gene expression profiles of *mas5Δ* (in blue) or *ssa2Δ* (in magenta) cells and wild type cells exposed to heat stress. Individual dots represent individual genes. Pearson's correlation coefficients and statistical significance are indicated. Venn diagrams (bottom panel) analyze the overlap in >2-fold differentially regulated genes between *mas5Δ* (in blue), *ssa2Δ* (in magenta) cells and heat-stressed wild type cells (in gray). (**B**) Comparison between gene expression profiles of *mas5Δ*, *ssa2Δ* and wild type cells under indicated environmental stresses; gene clustering presented on the left. (**C**) Expression profiles of genes specifically upregulated by distinct environmental stresses in *mas5Δ* and *ssa2Δ* cells and wild type cells under indicated environmental stresses. (**D**) Analysis of expression of common environmental stress response genes in exponentially growing *mas5Δ* and *ssa2Δ* cells, and *sty1Δ*, *atf1Δ* and wild type cells under indicated conditions. (**E**) Left panel shows the comparison between gene expression profiles of wild type heat-stressed cells, *mas5Δ*, *ssa2Δ* and cells overexpressing Hsf1, as detected by RNAseq analysis. Venn diagrams (right panel) analyze the overlap in >2-fold differentially regulated genes between *mas5Δ* (in blue), *ssa2Δ* (in magenta) cells, heat-stressed wild type cells (in gray) and cells overexpressing Hsf1 (in green).

We investigated the gene expression profiles of *mas5Δ*, *ssa2Δ* and heat-stressed wild type *S. pombe* cells by RNAseq analysis (See Materials and Methods for experimental details). Interestingly, we observed a striking similarity between their gene expression programs - majority of genes that >2-fold up- or down-regulated in the chaperone mutants exhibited similar regulation in heat-stressed cells (Fig. 6A).

Our RNAseq-based profiling of heat stress exhibited remarkable overlap with the microarray data on different abiotic stresses [62] allowing direct comparison between two technology platforms (Fig. S6A). The correlation between expression profiles in *mas5Δ* and *ssa2Δ* strains and wild type cells under distinct stress conditions confirmed similarities between expression profiles of the chaperone mutants and wild type cells exposed to heat and also suggested some similarities to cadmium-treated cultures (Fig. 6B and Fig. S6B–C). Gene expression linkage analysis identified a cluster enriched in genes reported as exclusively induced or super-induced in response to heat stress (Fig. 6B, red cluster, p = 2.65⋅10^−7^) [62]. Moreover, when we considered genes distinctly induced by different abiotic stresses, only the heat stress-specific genes were consistently upregulated in *mas5Δ* and *ssa2Δ* cells (Fig. 6C, p*^mas5Δ^* = 1.44⋅10^−2^, p*^ssa2Δ^* = 5.25⋅10^−6^ for 1.5-fold cut-off). No significant overlaps were detected for genes specifically up-regulated by other types of stress (Fig. 6C; p^binomial test^>0.25 for oxidative, osmotic stress and cadmium treatment). The microarray and qPCR analysis confirmed that genes specifically induced in response to heat stress were upregulated in *mas5Δ* cells and genes downregulated during heat-stress exhibited similar behavior in *mas5Δ* cells (Fig. S6D; See Supplementary Table S5 for detailed microarray analysis). We concluded that the transcriptional profiles of the chaperone mutants qualitatively matched the heat stress gene expression signature.

Both *mas5Δ* and *ssa2Δ* mutants exhibited differential regulation of most common environmental stress response (CESR) genes (Fig. 6D and [62]). Stress response is activated in part by MAP kinase (MAPK) signaling through the MAP kinase Sty1 and Atf1 transcription factor [59], [68], [69]. Interestingly, the most consistently up- or down-regulated CESR gene clusters were those independent of either Sty1 and/or Atf1 (Fig. 6D, clusters 4 and 6; Fig. S6E). While many Sty1- and Atf1-dependent CESR genes were also differentially regulated in the chaperone mutants (Fig. 6D), we failed to observe synthetic genetic interactions between *mas5Δ* and cells lacking Sty1 [70] or between *mas5Δ* and mutants carrying the constitutively activated MAPK kinase allele *wis1.DD* [59]. The phenotype of *mas5Δ* cells was also unchanged in the presence of Polo kinase mutant alleles *plo1S402A* and *plo1S402E* known to be defective in some aspects of the MAPK signaling [55].

We next wondered how the gene expression programs of heat-stressed cells and the chaperone mutants compared to that of cells overexpressing Hsf1. To this end, we performed RNAseq analysis of *nmt1:hsf1* cells grown in the absence of thiamine for 20 hours, at a time point when most Hsf1-overexpressing cells exhibited monopolar growth (Fig. 3). We observed a significant overlap between the sets of genes regulated by Hsf1 overexpression and the heat stress (Fig. 6E). Importantly, almost half of the differentially expressed genes in *mas5Δ* and/or *ssa2Δ* cells were similarly regulated in cells overexpressing Hsf1 (Fig. 6E). Many of those genes appeared the heat stress-responsive Hsf1-targets (Fig. S6F). It is possible that genes up-/down-regulated in the chaperone mutants that were not affected by Hsf1 overexpression could be regulated through other pathways. Alternatively, overexpression of Hsf1 could fail to fully recapitulate its native post-transcriptional activation.

Taken together, our gene expression analyses imply that cells lacking the Mas5-Ssa2 chaperone complex exhibit elevated levels of heat stress-associated transcription, at least in part due to abnormal activation of Hsf1.

### High Hsf1 activity accounts for the growth pattering defects in cells lacking Ssa2-Mas5 chaperone complex

**Figure 7:**
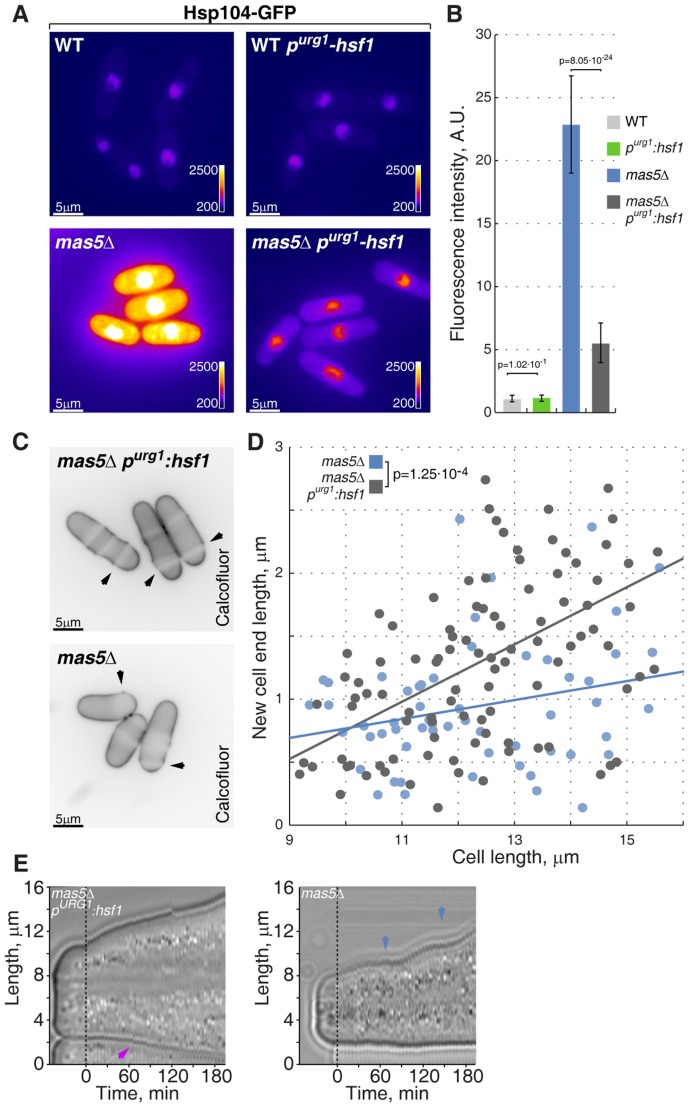
Repression of Hsf1 expression in *mas5Δ* cells rescues the growth patterning defects. (**A**) Hsp104-GFP expressing wild type and *mas5Δ* cells where Hsf1 expression was under the regulation of the native or inducible p^urg1^ promoter were grown in minimal medium lacking uracil for 36 hours at 24°C. Shown are pseudocolored epifluorescence images. Image contrast is reported using color wedges. Scale bars, 5 µm. (**B**) Quantification of GFP fluorescence in cells presented in (**A**). (**C**) Single z-plane images of calcofluor stained log-phase cells with indicated genotypes grown as described in (**A**). Arrowheads point to birth-scars. (**D**) New-cell-end length as a function of total cell length for cells with indicated genotypes. Points represent individual cells, lines represent the linear regression of a sample and the p-value was obtained through ANCOVA. (**E**) Kymographs of bright-field microscopy time-lapse analyses of cells with the indicated genetic backgrounds grown in a perfusion chamber at room temperature. Blue arrowheads point to growth pauses and a magenta arrowhead indicates onset of bipolar growth.

We wondered if polarized growth defects observed in the chaperone mutants were in fact related to high Hsf1 activity. To test this hypothesis, we sought to down-regulate Hsf1 expression in *mas5Δ* cells by promoter replacement. The *nmt81-hsf1* transcriptional shut-off allows full depletion of Hsf1 but the strains carrying this construct do not germinate and therefore are not suitable for genetic analyses (Fig. S3A and data not shown). Thus we replaced the native *hsf1* promoter with a recently reported uracil-regulatable p*^urg1^* promoter [71]. Repression of the p*^urg1^*-driven Hsf1 in wild type cells did not lead to growth arrest, suggesting that even in its repressed, basal state, p*^urg1^* promoter allows Hsf1 expression sufficient for supporting vegetative growth. In line with this, we did not observe differences in the basal levels of Hsp104-GFP reporter upon repression of *p^urg1^-hsf1* in unstressed wild type cells (Fig. 7A–B). However, Hsp104-GFP levels were strongly reduced in *mas5Δ* cells when the p*^urg1^*-driven Hsf1 expression was repressed (Fig. 7A–B).

Importantly, down-regulation of Hsf1 largely corrected the growth patterning defects of the chaperone mutant cells. Calcofluor staining revealed a significant increase in bipolar growth in *mas5Δ* cells with repressed *p^urg1^-hsf1* expression as compared to those carrying the wild type *hsf1* allele (Fig. 7C–D). Curiously, down-regulation of Hsf1 also rescued the intermittent growth phenotype, with *mas5Δ p^urg1^-hsf1* cells demonstrating continuous tip extension when the promoter was repressed (Fig. 7E; 12/20 *mas5Δ p^urg1^-hsf1* cells sustained continuous tip extension *vs* 5/20 *mas5Δ* cells).

Taken together, our results indicate that high Hsf1 activity leads to downregulation of Cdc42 module resulting in inability to support normal bipolar growth. Following stress-triggered Hsf1 activation, the Hsp70-Hsp40 chaperone pair Ssa2 and Mas5 could function to repress it, thus allowing cells to reset their transcriptional output and return to normal growth.

## Discussion

Organisms rapidly adjust their physiology in response to environmental stimuli such as changes in nutrient and mate availability, injury and abiotic stresses. A variety of stresses, including heat and exposure to osmolytes, trigger transient depolarization of the actin cytoskeleton and the cell wall remodeling machinery [25], [50], [53], [55]. After a period of adaptation to new conditions, the polarized growth is restored (Fig. 1 and S1). Our work focuses on this latter phase of stress response when cells resume polarized growth in an altered environment.

Following heat stress, the GTP-bound, active Cdc42 is no longer confined to the cell poles and redistributes throughout the entire cortex (Fig. 2). Cdc42 functions at the vertex of cellular growth (reviewed in [23], [72]) so its mis-localization likely propagates down to the cell wall remodeling machinery by delocalizing exocyst and the actin cytoskeleton (Fig. 1 and S1). Redistribution of the growth machinery throughout the cortex has been proposed to function in strengthening the cell wall and possibly to fix injuries caused by a temperature increase [49], [73], [74], although we did not readily detect major changes in cell wall appearance in heat-stressed *S. pombe* cells by electron microscopy (data not shown). The reported diffusion rates of the membrane-bound Cdc42 are relatively low [75] suggesting that mis-localization is likely due to Cdc42 activation occurring throughout the cortex rather than the lateral movement of active GTPase from the cell tips. Overexpression of Gef1 is sufficient to promote Cdc42 activation throughout the entire plasma membrane [76] suggesting that modulation of Gef1 activity and/or membrane recruitment upon heat stress could allow for ectopic activation of Cdc42. Interestingly, inhibition of the evolutionary conserved NDR kinase Orb6 promotes invasion of Cdc42 into the lateral cortical domain possibly through direct phosphorylation of Gef1 [37]. Activity of GEFs could be also modulated by interaction with plasma membrane proteins sensing the environment or the structural integrity of the cell wall. For instance, in budding yeast the cell wall sensors Wsc1 and Mid2 have been shown to interact with the GEF for Rho1, Rom2, and stimulate the Rho1 nucleotide exchange [51]. Since all landmark proteins including Pom1, the direct modulator of Rga4/Cdc42-GAP activity [39], remain properly polarized during heat stress response (Fig. 1), the mechanisms regulating delocalization of active Cdc42 are likely to function independently of the polarisome.

Once the initial phase of stress response is over, cells must return to normal growth in new conditions. Curiously, transient depolarization of the growth machinery upon mild heat stress is followed by a prolonged period of monopolar growth ([21], Fig. 1). Since the entire cell population becomes predominantly monopolar it means that heat stress could delay both the onset of NETO in newly born cells and re-initiation of persistent bipolar growth in late G_2_ phase of the cell cycle. The predominantly monopolar growth pattern in cells adapting to heat stress could be possibly explained by the recently developed model correlating the cell length to the bipolar Cdc42 activation [40]. The salient prediction of the model is that reduction of the positive relative to the negative Cdc42 feedback would result in an increased incidence of monopolar cells. Reduction in cortical levels of Cdc42-GEFs in heat-stressed cells could indeed weaken the positive feedback [42], [77], [78]. It is known that monopolar growth does result from the perturbed regulation of Cdc42 activity. *S. pombe* cells lacking Cdc42-GEF Gef1 have been shown to delay NETO [76]. Similarly, mutants in the Cdc42 effector PAK kinase that likely functions as a part of Cdc42 activity feedback [40] are monopolar [79]. The nature of negative feedback is less clear but it is unlikely to be limited to Cdc42-GAP [40], [41]. Alternatively, the heat-induced chaperones could directly modulate the cytoskeletal dynamics [80], [81] and in turn, affect the Cdc42 module activity.

Of note, cells with high levels of heat stress associated transcription either due to overexpression of the heat shock transcription factor Hsf1 or lacking the chaperone complex Ssa2-Mas5, also grow in a monopolar fashion and exhibit decreased cortical levels of active Cdc42 (Fig. 3 and Fig. 5). Unlike in a polarisome-deficient monopolar mutant *tea1Δ*, the active Cdc42 can be recruited to both tips of *mas5Δ* cells (Fig. S5R). Yet, only the old cell end that was inherited from the mother is capable of maintaining Cdc42 activity levels sufficient for growth (Fig. 5F). Consistent with an idea that low Cdc42 activity does not allow bipolar growth in cells where the heat stress response is on, mild overexpression of the constitutively active Cdc42 mutant rescued the growth patterning defects of *mas5*Δ cells (Fig. 5G–I).

Once wild type cells attain the new steady state growth at elevated temperature, the cortical activity of Cdc42 returns to normal and the bipolar growth is resumed (Fig. S2). Interestingly, the abundance of Cdc42 regulators is inversely correlated with environmental temperature (Fig. S2H), which may suggest a degree of temperature compensation in the Cdc42 module. The eukaryotic circadian clock is a typical example of a complex feedback network that buffers the period of oscillations against temperature (reviewed in [82]). The temperature compensation of the *Neurospora* clock relies on phosphorylation of the key regulator Frq by the casein kinase 2 [83], an ortholog of the gene isolated as *orb5* in the fission yeast morphogenesis screen [84]. Furthermore, Hsf1 itself is known to regulate temperature compensation of the mammalian circadian clock [85]. The decrease in the Cdc42 activity in cells lacking the Ssa2-Mas5 complex could be potentially explained by over-compensation of Cdc42 module in the absence of the actual temperature increase.

Our work suggests that in fission yeast, the Hsp40 protein Mas5 functions in repressing Hsf1 activity as a specificity co-factor for the Hsp70 chaperone Ssa2. Heat shock transcription factors are extensively regulated [63] and both Hsp90 and Hsp70 chaperones have been proposed to function as their repressors [18], [19], [86]. We show that transcriptional depletion of Hsf1 indeed suppresses the heat-stress associated transcription in *mas5*Δ cells and also rescues the polarized growth defects in this chaperone mutant (Fig. 7). In line with this, the slow growth of budding yeast lacking Ssa1 and Ssa2 is rescued by a partial loss-of-function mutation in Hsf1 [87]. An interesting twist to the chaperone-mediated regulation is that mRNA abundance of these chaperones increases upon heat stress (1.9-fold and 3.3-fold increase, respectively), possibly placing Hsf1 activity within a negative feedback loop. The cytosolic Hsp70 chaperones could also act as primary sensors of stress at least in some conditions, *e. g.* Ssa1 in budding yeast has been shown to release Hsf1 from the inhibitory complex upon binding to the thiol-reactive compounds [88]. The human Mas5 and Ssa2 orthologs have been shown to bind Hsf1 *in vitro* [89], an interaction that we were unable to detect in fission yeast, possibly due to its transient nature.

Several reports suggested that subcellular distribution of Hsf1 could affect its activity (surveyed in [90]). Interestingly, we found that during the steady state growth in *S. pombe*, Hsf1 exhibited a predominantly nuclear localization but it was fully exported into the cytoplasm following heat stress (Fig. S7). However, in the absence of Mas5 and Ssa2 Hsf1 failed to exit the nuclei of heat-stressed cells, suggesting that the chaperone complex could also regulate Hsf1 through controlling its subcellular distribution (Fig. S7). The temporally resolved studies of Hsf1-driven transcription triggered by heat stress suggest that the initial burst of Hsf1 activity is rapidly followed by its repression [6], [8], [62], [91]. Removing Hsf1 from the nucleus could be one of the mechanisms to attenuate its activity.

The Mas5-like Hsp40 chaperones are highly promiscuous in their interactions with client proteins [65]. This property could potentially allow them to serve as sensors of global folding status linking the machineries handling environmental stress response and the normal cellular growth. Indeed, Ydj1, the budding yeast ortholog of Mas5, has been proposed to function as a growth rate sensor regulating G_1_/S entry [92], [93]. Curiously, bursts of fairly normal tip extension in individual *mas5*Δ or *ssa2*Δ cells are interspersed with periods of rest (Fig. 5), suggesting that one or more factors could become limiting for growth when availability of these chaperones is decreased. Given that the instantaneous growth rate in yeast has been negatively correlated to upregulation of heat-responsive genes [94], the Hsf1-chaperone interaction could function in a feedback loop sustaining the normal cellular growth. It is worth noting that while both upregulation of Cdc42 activity and partial downregulation of Hsf1 in the chaperone mutant cells rescued the polarity-related phenotypes, neither genetic manipulation ameliorated the slow doubling time of *mas5*Δ cells.

Placing the central transcriptional activator of the heat shock response under negative regulation by chaperones with a wide client base may ensure efficient and highly sensitive means of adapting to frequently changing environment. Moreover, this circuitry appears to be coopted in regulating the normal cell cycle progression as a function of growth rate. By regulating Hsf1 activity through the Ssa2-Mas5 chaperone system, fission yeast cells could optimize Cdc42-driven polarized growth under different temperature regiments. Given that the heat stress associated transcription modulates diverse cellular functions [1], similar adaptive dynamics are likely to operate in other physiological processes.

## Materials and Methods

### *S. pombe* strains and reagents

*S. pombe* strains used in this study and their genotypes are listed in Supplementary Table S1. Growth media and genetic methods were as described in [95]. The actin marker Lifeact-GFP was a kind gift from M. Balasubramanian. The cell wall dyes Calcofluor White and FITC-Lectin were obtained from Sigma-Aldrich (St. Louis, MO, USA). We placed GFP under the control of the *hsp104* promoter region (1.5 kb upstream of the start codon) and the 3′ UTR (0.7 kb downstream of the stop codone) to construct a reporter of heat-activated expression. The construct was integrated into *hsp104* chromosomal locus without disrupting the Hsp104 ORF. For Hsf1 overexpression and thiamine-responsive transcriptional shut-off we replaced the *hsf1* promoter with *nmt1* and *nmt81* promoters respectively at the native chromosomal locus. Similarly, for uracil-responsive modulation of Hsf1 expression we replaced the *hsf1* promoter with *urg1* promoter (675 bp upstream of the start codon) at the native chromosomal locus.

### Fluorescence Activated Cell Sorting (FACS)

FACS was performed on MACSQuant Analyzer (Miltenyi Biotec GmbH, Bergisch Gladbach, Germany) using a protocol described by [96] with RNase obtained from Roche (Basel, Switzerland) and propidium-iodide from Sigma-Aldrich (St. Louis, MO, USA).

### Microscopy

Epifluorescence images were collected using mercury lamp as an illumination source with appropriate sets of filters on a Zeiss Axiovert 200M microscope (Carl Zeiss, Inc.) using an Plan Apochromat 100X, 1.4 N.A. objective lens, CoolSnap camera (Photometrics, Tucson, AZ) and Uniblitz shutter driver (Photonics, Rochester, NY) under the control of Metamorph software package (Universal Imaging, Sunnyvale, CA). We acquired either single z-planes or whole cell image stacks that consisted of 9 z-sections with 0.5 µm spacing. The z-stack maximum or average projection images were obtained by ImageJ 1.46b software package (http://rsb.info.nih.gov/ij/; National Institutes of Health, Bethesda, MD, USA). The same setup was also used in DIC microscopy on fission yeast cells grown in liquid YES medium using ONIX perfusion chambers (CellASIC, Hayward, CA, USA) under the control of the proprietor software and the flow of 4 psi.

Scanning confocal microscopy was performed on a LSM510 microscope equipped with a Plan Apochromat 100X, 1.4 N.A. objective lens, a 488-nm argon laser and a 543-nm HeNe laser. We acquired either single z-planes or whole cell image stacks that consisted of 9 z-sections with 0.5 µm spacing. The z-stack maximum or average projection images were obtained by ImageJ 1.46b software package.

Time-lapse fluorescence microscopy images were generated on Nikon TiE system (CFI Plan Apochromat VC 100XH 1.4 N.A. objective) equipped with Yokogawa CSU-X1-A1 spinning disk unit, the Photometrics CoolSNAP HQ2 camera and a DPSS 491 nm 100 mW and DPSS561 nm 50 mW laser illumination under the control of MetaMorph Premier Ver. 7.7.5. We acquired single z-plane images. Imaging was performed on *S. pombe* cells placed in sealed growth chambers containing 2% agarose YES medium.

### Image analyses

Longitudinal growth in lectin stained cells was assessed manually as a distance from the cell tip to the lectin signal along the long cell axis.

New-cell-end growth was quantified from calcofluor-stained cells as the distance from the birth scar to the proximal cell tip and expressed as a function of total cell length. We employed ANCOVA analysis to assess the statistical significance of variation in new-end growth between cells with different genotypes (n>30 cells per genotype). For simplified presentation we also estimated average new-end length in cells 12–15 µm long while making sure that the average cell length between samples was comparable, p-values were obtained using Welch's t-test.

ImageJ in-built line-scan module was used to create an intensity profile along the long axis of the cells expressing CRIB-GFP. The positional information was normalized as the percentage of the cell length. Cellular fluorescence profiles were calculated from background-subtracted images and the nuclear signal was set as one arbitrary unit since it did not vary significantly between samples. In Fig. 2C unscaled fluorescence intensities are reported and were obtained from cells with the same levels of total CRIB-GFP fluorescence.

Fluorescence levels of Cdc42 regulators were analyzed from background subtracted images by manually outlining the whole cells and cell tips. Reported relative changes in fluorescence intensities are based on the average calculated values.

ImageJ 1.46b software package was used for all image quantifications.

### Total protein extraction, co-immunoprecipitation

For co-immunoprecipitation, yeast cells were grown to log phase, harvested and washed with Buffer A (6 mM Na2HPO4, 4 mM NaH2PO4.H2O, 150 mM NaCl, 2 mM EDTA, 50 mM NaF, 0.1 mM Na3VO4, protease inhibitor cocktail (Roche)). Cell pellets were resuspended in 200 µL of buffer A, mixed with glass beads and homogenized in a Mini Bead Beater (Biospec, Bartlesville, OK, USA) at 4°C. Total cell lysate were harvested and centrifuged (16,000× g, 10 min) to remove cell debris. Soluble fractions were adjusted to same total protein concentration using Buffer A and 350 µl were incubated with either mouse *anti*-myc (Milipore, Billerica, MA, USA) antibodies and Protein A beads (Invitrogen, Carlsbad, CA, USA) or just GFP-Trap beads (ChromoTek, Munich, Germany) for 1 hour. Beads were washed 6 times with 1 mL of buffer A and resuspended in 50 µL of SDS-Loading buffer.

For total protein quantifications, cells were pelleted and resuspended in 200 µl of BufferA (1.85M NaOH, 1M β-mercaptoethanol, 5× EDTA-free Complete Protease Inhibitor Cocktail (Roche)) and kept on ice for 10 min. We added 450 µl of water and 350 µl of 6.1N trichloroacetic acid. The samples were incubated in ice for 10 min and centrifuged for 10 min at 4°C. The obtained pellet was washed with 0.5 M Tris-base, dissolved in gel loading buffer and kept at 65°C for 10 min and 95°C for 3 min.

Protein samples were subjected SDS-PAGE and standard Western Blotting. Proteins of interest were probed by mouse *anti*-GFP (Roche), rabbit *anti*-HA (Roche) and mouse *anti*-myc (Milipore). Rabbit *anti*-HistoneH3 (Abcam, Cambridge, MA, USA) probing served to monitor sample loading. IRDye800 conjugated *anti*-mouse and IRDye700 conjugated *anti*-rabbit antibodies were used prior to analysis on the Odyssey Infrared Imaging system, all purchased from LI-COR Biosciences (Lincoln, NE, USA).

### RNA sequencing (RNAseq) analyses

Cells were grown in standard YE (wild-type, *mas5*Δ, *ssa2*Δ) or MM media (wild type, *nmt1:hsf1* without thiamine, *nmt81:hsf1* with thiamine) at 24°C. A sample of wild type cells was also shifted from 24°C to 36°C for 15 minutes. RNA was extracted using Qiagen RNeasy Mini Kit (Qiagen, Venlo, Netherlands) following manufacturer's protocol. Samples were sequenced by single end 50 bp sequencing at the University of Utah's Bioinformatics and Microarray Next Generation Sequencing Shared Resources (Salt Lake City, Utah, USA) after Illumina TruSeq RNA Sample Prep with oligo-dT selection. The RNA sequencing results were analyzed using Galaxy software package [97]–[99]. Briefly, upon quality control using the FastQ Groomer [100] module, reads were mapped to the fission yeast genome using the Tophat module [101] and differential gene expression was assessed by the Cufflinks module [102]. Analyses of differential gene expression are presented in Supplementary Table S4. The relevant raw RNAseq data have been deposited in NCBI's Gene Expression Omnibus [103] and are accessible through GEO Series accession number GSE50156 (http://www.ncbi.nlm.nih.gov/geo/query/acc.cgi?acc=GSE50156).

### Quantitative PCR

The RNA samples from *mas5Δ* and wild type cells grown at 24°C or shifted to 36°C for 45 min were prepared using phenol-chlorophorm extraction described by [104] and reverse transcribed using SuperScript III (Invitrogen) and random decamer primers (N10) at 50°C for 60 min. The reverse transcriptase was inactivated at 70°C for 10 min. cDNA samples were analyzed by quantitative real-time PCR (qPCR) using a StepOnePlus real-time PCR system (Applied Biosystems) and Fast SYBR Green Master Mix (Applied Biosystems) with 0.15 µM of each forward and reverse primers. The following cycling program was used: 95°C for 20 s, followed by 45 cycles of a three-step reaction, denaturation at 95°C for 5 s, annealing and extension at 60°C for 45 s. The primers used are listed in Supplementary Table S3. Data shown were normalized to the expression levels of actin (*act1*) and similar results were obtained when using GAPDH (*gpd1*, data not shown) to normalize the data.

### Microarray analysis

The gene expression profile of *mas5Δ* cells was obtained as previously described by [104]. The significance of differential gene regulation between wild type and *mas5Δ* cell was assessed by the Welch's t-test (Supplementary Table S5). Only genes with p<0.05 and fold change >1.5 were included in subsequent analysis. The expression profiles for stress conditions were taken from [62].

Clustering analysis was performed using Gene Cluster [5] using uncentered correlation similarity metrics average-linkage method. The visualization was done using TreeView software [105].

Correspondence at the top plot was derived from [106]. Briefly, the genes were sorted into a list according to how pronounced their up/down-regulation was in *mas5Δ* as compared to wild type cells. The binomial test was then employed to generate p-values as a function of an increasing number of genes included into the comparison between *mas5Δ* expression profile and that of stressed cells.

## Supporting Information

Figure S1 Heat stress in fission yeast leads to a transient loss of cell polarity succeeded by a phase of monopolar growth. (**A**) Single z-plane images of calcofluor stained log-phase wild type cells grown at 24°C (left panel) or 36°C (right panel) for 20 hours. Arrowheads point to birth-scars. Note that cells in cultures shifted to 36°C eventually regain bipolarity. (**B–E**) Shown are whole cell maximum intensity *z*-projections of confocal micrographs of log-phase wild type cells expressing indicated fluorophore-tagged marker proteins grown at 24°C (left panels) or shifted to 36°C for 45 min (middle panels) or 105 min (right panels). The arrowheads point out localization of the marker proteins at the lateral cell cortex. Image contrast is reported using corresponding gray wedges. Scale bars, 5 µm. (**F**) Quantification of late G_2_ cells with monopolar distribution of indicated marker proteins in cells grown at 24°C (gray) or shifted to 36°C for 105 min (blue). (**G**) Quantification of the imbalance in number of individual actin structures between localizing to two cell ends in late G_2_ cells grown at 24°C (gray) or shifted to 36°C for 105 min (blue). n>20 cells per sample.
(TIF)

Figure S2 Behavior of Cdc42 regulators is modulated by temperature. (**A**) Quantification of late G_2_ cells with monopolar distribution of indicated marker proteins in cells grown at 24°C (gray) or shifted to 36°C for 105 min (blue). (**B**) Wild type cells expressing Gef1-3YFP, Scd1-GFP or Rga4-GFP were grown under indicated conditions prior to total protein extraction. SDS-PAGE resolved protein samples were subjected to Western Blotting using *anti*-GFP antibodies (top panels). HistoneH3 probing (bottom panels) served as a sample loading control. (**C, E–G**) Cells expressing indicated fluorophore-tagged proteins were grown overnight at 18°C (left panels), 24°C (middle panels) or 30°C (right panels). All images shown are epifluorescence micrographs of log-phase cells. Gray wedges report the image contrasting. Scale bars, 5 µm. (**D**) Quantification of CRIB-GFP intensities along the long cell axis in log-phase wild type cells grown to log-phase at indicated temperatures. Individual lines correspond to individual cells. (**H**) Wild type cells expressing Gef1-3YFP, Scd1-GFP or Rga4-GFP were grown overnight to log-phase (O.D.^595^≈0.4) at indicated temperatures prior to total protein extraction. SDS-PAGE resolved protein samples were subjected to Western Blotting using *anti*-GFP antibodies (top panels). HistoneH3 probing (bottom panels) served as a sample loading control. Note that abundance of Rga4 is anti-correlated with environmental temperature.
(TIF)

Figure S3 Overexpression of Hsf1 elevates expression of heat-induced genes in fission yeast. (**A, B**) Both overexpression and depletion of Hsf1 lead to growth arrest as shown by these growth assays of cells with indicated genotypes in the absence (left panel) or presence (right panel) of thiamine in the growth media. Presence of thiamine represses the *nmt*-based promoters. *nmt81* is a weak promoter and is used for transcriptional shut-off of Hsf1 (left panel, note that cells do not grow in the presence of thiamine). *nmt1* is a strong promoter and is used for Hsf1 overexpression (right panel, note that cells do not grow in the absence of thiamine). (**C**) Hsp104-GFP fluorescence in cells with *hsf1* ORF under the control of the wild type or *nmt1* promoter grown to log phase in minimal medium without thiamine. Shown are pseudocolored average intensity whole cell *z*-projections of Hsp104-GFP epifluorescence. Note that Hsp104 abundance is increased in cells overexpressing Hsf1. Image contrast is reported using corresponding color wedges. Scale bars, 5 µm. (**D**) Quantification of Hsp104-GFP fluorescence in log-phase wild type cells grown at 24°C and shifted to 36°C for 45 min or 105 min (left panel) and between the wild type and *nmt1::hsf1* cells grown to log phase in absence of thiamine. (**E**) Quantification of GFP expression driven by the *hsp104* regulatory elements in log-phase wild type cells grown at 24°C and shifted to 36°C for 45 min or 105 min (left panel) and between the wild type and *nmt1::hsf1* cells grown to log phase in absence of thiamine.
(TIF)

Figure S4 Additional characterization of the chaperone deletion strains identified in screening for mutant cells with elevated levels of heat stress associated transcription. (**A**) Lysates prepared from cells with indicated genotypes were incubated with *anti*-myc antibodies and subsequently with beads coupled to protein-G. Proteins that remained associated with the beads after multiple buffer washes were resolved by SDS-PAGE and prepared for western blotting with *anti*-myc and *anti*-HA antibodies. (**B**) Pseudocolored average *z*-projection epifluorescence images of the Hsp104-GFP wild type, *ssa1Δ*, *ssa2Δ* and *mas5Δ* cells grown under indicated conditions. Image contrast is reported using corresponding color wedges and scale bars correspond to 5 µm. Top right panel histogram quantifies the fluorescence signal in indicated strains.
(TIF)

Figure S5 Cells lacking Mas5 or Ssa2 exhibit monopolar intermittent growth in G_2_ phase of the cell cycle. (**A**) OD^595 nm^ measurements of log-phase cell cultures of cells with indicated genotypes grown at 24°C (left panel) and average length at division at OD^595 nm^ = 0.5 (right panel). (**B**) FACS analysis of DNA content for log-phase wild type cells (black), wild type cells arrested in S phase using hydroxyurea (gray) and log-phase *ssa2Δ* (purple) and *mas5Δ* cells (blue). (**C**) Quantification of late G_2_ cells with monopolar distribution of indicated marker proteins in wild type (gray) and *mas5Δ* cells (blue). (**D–I, K–L**) Shown are whole cell maximum intensity *z*-projections of confocal micrographs of log-phase wild type (left panels) and *mas5Δ* (right panels) cells expressing indicated fluorophore-tagged marker proteins grown at 24°C. (**J**) Quantification of the imbalance in number of individual actin structures between localizing to both cell tips in wild type (in gray) and *mas5Δ* (in blue) late G_2_ cells grown at 24°C. n>20 per sample. (**M–O**) Shown are single *z*-plane micrographs of log-phase wild type (left panels) and *mas5Δ* (right panels) expressing indicated fluorophore-tagged marker proteins grown at 24°C (left panels). (**Q**) Single z-plane images of cells with indicated genotypes grown in minimal media in the absence of thiamine for 30 hours and shifted to complete media for 7 hours prior to imaging. Note that the dominant-active mutant Cdc42^G12V^, but not the wild type protein, is enriched at the cellular cortex. (**P**) Wild type and *mas5Δ* cells expressing indicated marker proteins were grown overnight to log-phase (OD^595 nm^≈0.4) at indicated temperatures prior to total protein extraction. SDS-PAGE resolved protein samples were subjected to Western Blotting using *anti*-GFP antibodies (top panels). HistoneH3 probing (bottom panels) served as a sample loading control. (**R**) Kymographs of single *z*-plane spinning disk confocal microscopy time-lapse analyses of wild type (top panel), *mas5Δ* (middle panel) and *tea1Δ* cells (bottom panels) grown at room temperature. Arrowheads point to recruitment of active Cdc42 to the new cell tip.
(TIF)

Figure S6 Cells lacking the Ssa2-Mas5 chaperone complex exhibit elevated levels of heat-stress associated transcription. (**A**) Right panel represents correlation analysis between gene expression profiles of wild type heat-stressed cells analyzed by RNAseq and microarray technologies. Individual dots represent individual genes. Pearson's correlation coefficients and statistical significance are indicated. Venn diagrams analyze the overlap in genes differentially regulated >2-fold (in red), >1.5-fold (in yellow) obtained via microarray (Chen *et al.*, 2003) and >2-fold according to RNAseq (in gray). Correlation analysis between gene expression profiles of *mas5Δ* (**B**) or *ssa2Δ* (**C**) cells and wild type cells under indicated environmental stresses. Individual dots represent individual genes. (**D**) Expression levels of genes specifically induced and genes repressed during heat-stress in wild type cells grown at 24°C, shifted to 36°C for 45 min and *mas5Δ* cells as measured by qPCR. Expression levels are normalized to wild type cells grown at 24°C. (**E**) Venn diagrams analyze the overlap in genes differentially regulated >2-fold in *mas5Δ* or *ssa2Δ* cells and in heat-stressed *sty1Δ* or atf1*Δ* cells (Chen *et al.*, 2003). The top set of panels is based on a >2-fold cut-off for data on *sty1Δ* or atf1*Δ* cells. The top set of panels is based on a >1.5-fold cut-off for data on *sty1Δ* or atf1*Δ* cells. (**F**) Venn diagrams analyze the overlaps between the gene sets that are differentially regulated >2-fold in *mas5*Δ or *ssa2*Δ cells and the heat stress-responsive Hsf1-dependent genes.
(TIF)

Figure S7 Cells lacking the Ssa2-Mas5 chaperone complex exhibit impaired nucleocytoplasmic shuttling of Hsf1. Shown are whole cell maximum intensity *z*-projections of scanning confocal micrographs of log-phase cells with indicated genotypes expressing GFP-Hsf1 from its native promoter grown at 24°C (left panels) or shifted to 36°C for 45 min (right panels). Image contrast is reported using corresponding color wedges. Scale bars, 5 µm.
(TIF)

Movie S1 Cells lacking Mas5 and Ssa2 exhibit monopolar intermittent growth. Cells with indicated genotypes were grown to log phase and shifted to ONIX perfusion chambers for long-term imaging. Time indicated is in hours∶minutes format.
(MOV)

Table S1 List of *Schizosaccharomyces pombe* strains used in this study.
(DOCX)

Table S2 List of strains used in the screening for cells with elevated levels of heat stress-associated transcription.
(DOCX)

Table S3 List of primers used in the qPCR analysis of differential gene expression in heat-stressed wild type and *mas5*Δ cells.
(DOCX)

Table S4 RNAseq results and analyses of differential gene expression in *mas5*Δ, *ssa2*Δ, heat-stressed wild type cells and cells overexpressing Hsf1.
(XLSX)

Table S5 Microarray results and analyses of differential gene expression in *mas5*Δ and wild type cells grown at 24°C.
(XLS)
