# Supplementary figures and images for: Hsp70-Hsp40 Chaperone Complex Functions in Controlling Polarized Growth by Repressing Hsf1-Driven Heat Stress-Associated Transcription
Source: PLoS Genet. 2013 Oct 17;9(10):e1003886. doi: 10.1371/journal.pgen.1003886 (PMC3798271; doi:10.1371/journal.pgen.1003886)

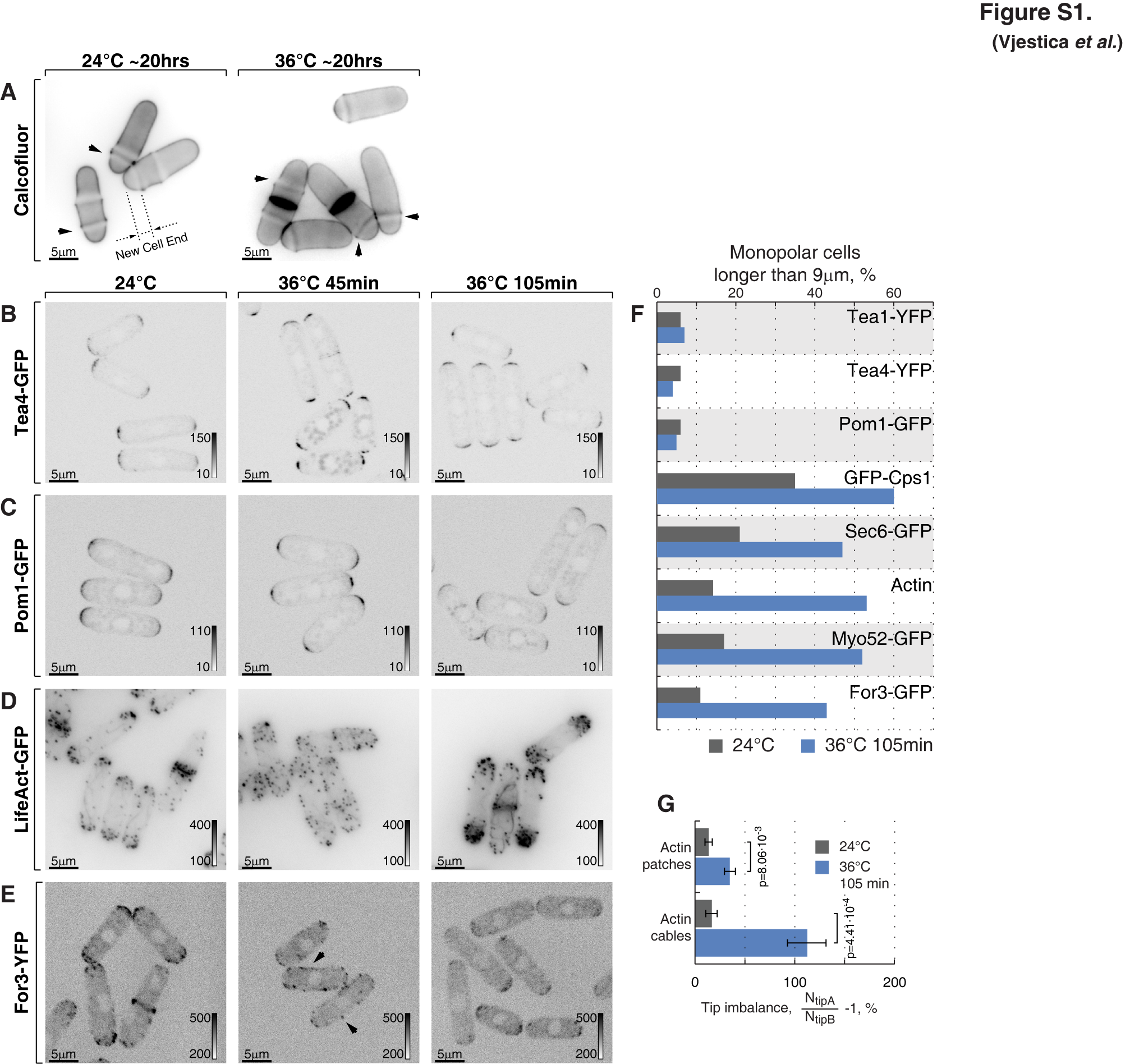

Supplement: Figure S1 — Heat stress in fission yeast leads to a transient loss of cell polarity succeeded by a phase of monopolar growth. (A) Single z-plane images of calcofluor stained log-phase wild type cells grown at 24°C (left panel) or 36°C (right panel) for 20 hours. Arrowheads point to birth-scars. Note that cells in cultures shifted to 36°C eventually regain bipolarity. (B–E) Shown are whole cell maximum intensity z-projections of confocal micrographs of log-phase wild type cells expressing indicated fluorophore-tagged marker proteins grown at 24°C (left panels) or shifted to 36°C for 45 min (middle panels) or 105 min (right panels). The arrowheads point out localization of the marker proteins at the lateral cell cortex. Image contrast is reported using corresponding gray wedges. Scale bars, 5 µm. (F) Quantification of late G2 cells with monopolar distribution of indicated marker proteins in cells grown at 24°C (gray) or shifted to 36°C for 105 min (blue). (G) Quantification of the imbalance in number of individual actin structures between localizing to two cell ends in late G2 cells grown at 24°C (gray) or shifted to 36°C for 105 min (blue). n>20 cells per sample. (TIF) [file pgen.1003886.s001.tif]

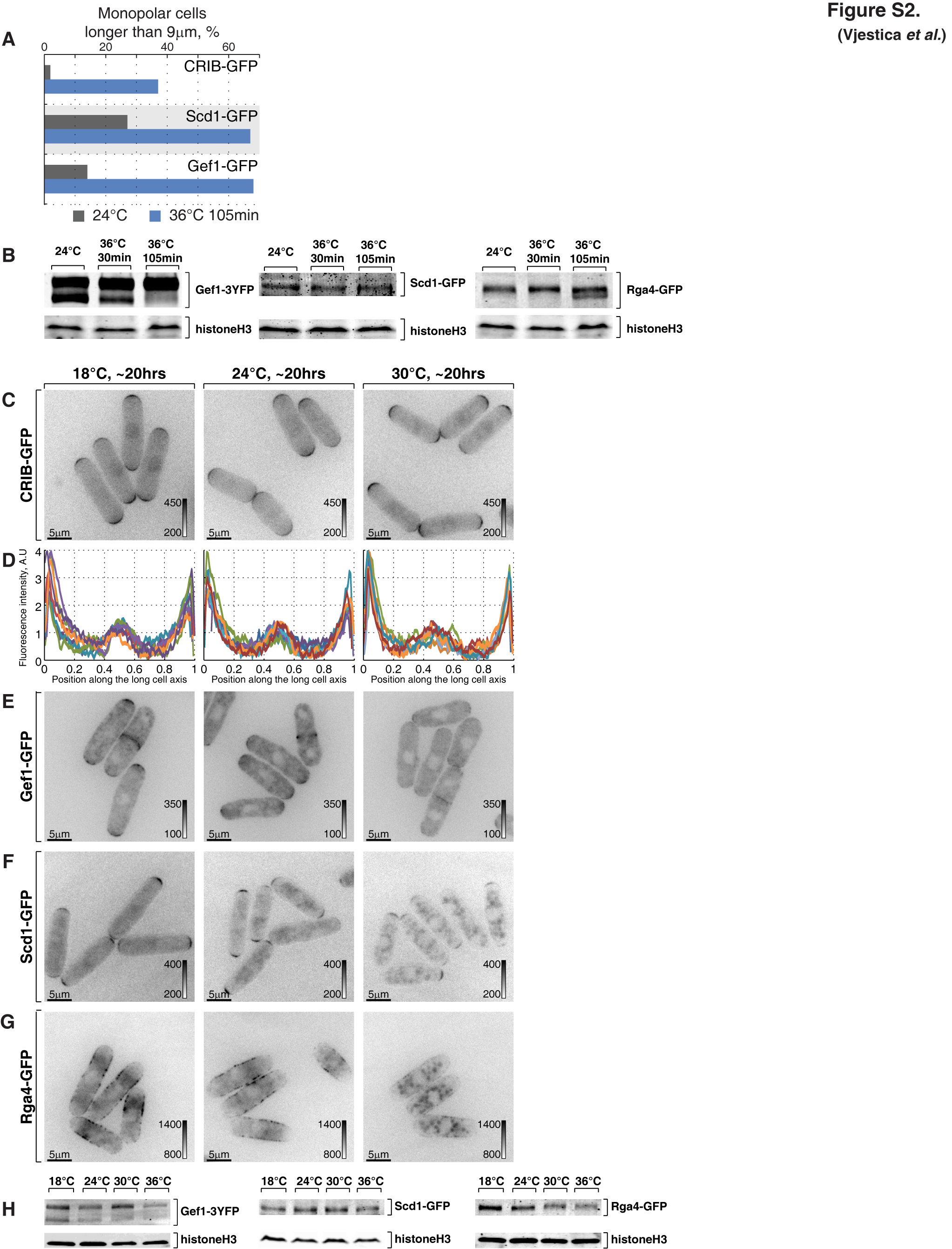

Supplement: Figure S2 — Behavior of Cdc42 regulators is modulated by temperature. (A) Quantification of late G2 cells with monopolar distribution of indicated marker proteins in cells grown at 24°C (gray) or shifted to 36°C for 105 min (blue). (B) Wild type cells expressing Gef1-3YFP, Scd1-GFP or Rga4-GFP were grown under indicated conditions prior to total protein extraction. SDS-PAGE resolved protein samples were subjected to Western Blotting using anti-GFP antibodies (top panels). HistoneH3 probing (bottom panels) served as a sample loading control. (C, E–G) Cells expressing indicated fluorophore-tagged proteins were grown overnight at 18°C (left panels), 24°C (middle panels) or 30°C (right panels). All images shown are epifluorescence micrographs of log-phase cells. Gray wedges report the image contrasting. Scale bars, 5 µm. (D) Quantification of CRIB-GFP intensities along the long cell axis in log-phase wild type cells grown to log-phase at indicated temperatures. Individual lines correspond to individual cells. (H) Wild type cells expressing Gef1-3YFP, Scd1-GFP or Rga4-GFP were grown overnight to log-phase (O.D.595≈0.4) at indicated temperatures prior to total protein extraction. SDS-PAGE resolved protein samples were subjected to Western Blotting using anti-GFP antibodies (top panels). HistoneH3 probing (bottom panels) served as a sample loading control. Note that abundance of Rga4 is anti-correlated with environmental temperature. (TIF) [file pgen.1003886.s002.tif]

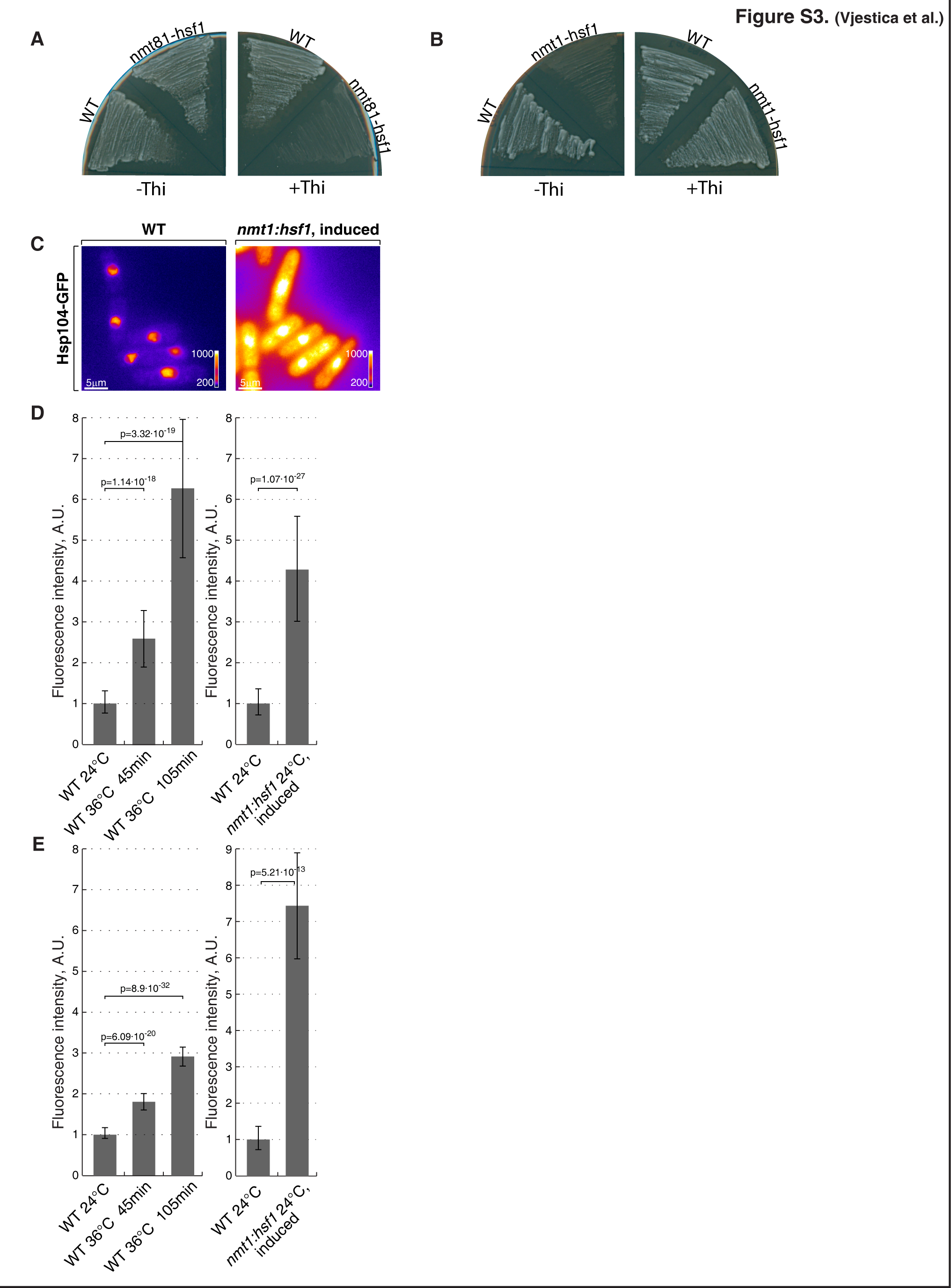

Supplement: Figure S3 — Overexpression of Hsf1 elevates expression of heat-induced genes in fission yeast. (A, B) Both overexpression and depletion of Hsf1 lead to growth arrest as shown by these growth assays of cells with indicated genotypes in the absence (left panel) or presence (right panel) of thiamine in the growth media. Presence of thiamine represses the nmt-based promoters. nmt81 is a weak promoter and is used for transcriptional shut-off of Hsf1 (left panel, note that cells do not grow in the presence of thiamine). nmt1 is a strong promoter and is used for Hsf1 overexpression (right panel, note that cells do not grow in the absence of thiamine). (C) Hsp104-GFP fluorescence in cells with hsf1 ORF under the control of the wild type or nmt1 promoter grown to log phase in minimal medium without thiamine. Shown are pseudocolored average intensity whole cell z-projections of Hsp104-GFP epifluorescence. Note that Hsp104 abundance is increased in cells overexpressing Hsf1. Image contrast is reported using corresponding color wedges. Scale bars, 5 µm. (D) Quantification of Hsp104-GFP fluorescence in log-phase wild type cells grown at 24°C and shifted to 36°C for 45 min or 105 min (left panel) and between the wild type and nmt1::hsf1 cells grown to log phase in absence of thiamine. (E) Quantification of GFP expression driven by the hsp104 regulatory elements in log-phase wild type cells grown at 24°C and shifted to 36°C for 45 min or 105 min (left panel) and between the wild type and nmt1::hsf1 cells grown to log phase in absence of thiamine. (TIF) [file pgen.1003886.s003.tif]

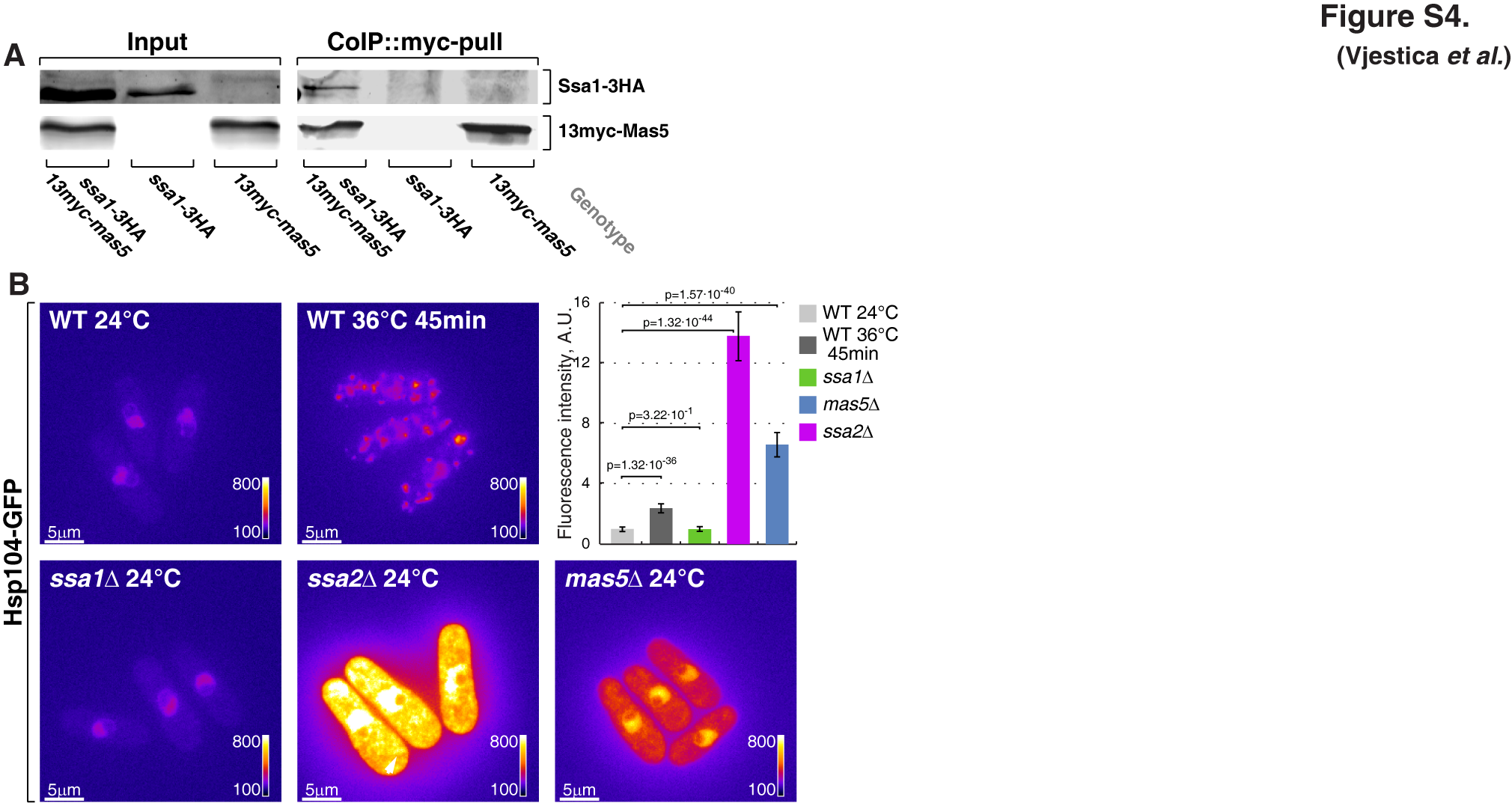

Supplement: Figure S4 — Additional characterization of the chaperone deletion strains identified in screening for mutant cells with elevated levels of heat stress associated transcription. (A) Lysates prepared from cells with indicated genotypes were incubated with anti-myc antibodies and subsequently with beads coupled to protein-G. Proteins that remained associated with the beads after multiple buffer washes were resolved by SDS-PAGE and prepared for western blotting with anti-myc and anti-HA antibodies. (B) Pseudocolored average z-projection epifluorescence images of the Hsp104-GFP wild type, ssa1Δ, ssa2Δ and mas5Δ cells grown under indicated conditions. Image contrast is reported using corresponding color wedges and scale bars correspond to 5 µm. Top right panel histogram quantifies the fluorescence signal in indicated strains. (TIF) [file pgen.1003886.s004.tif]

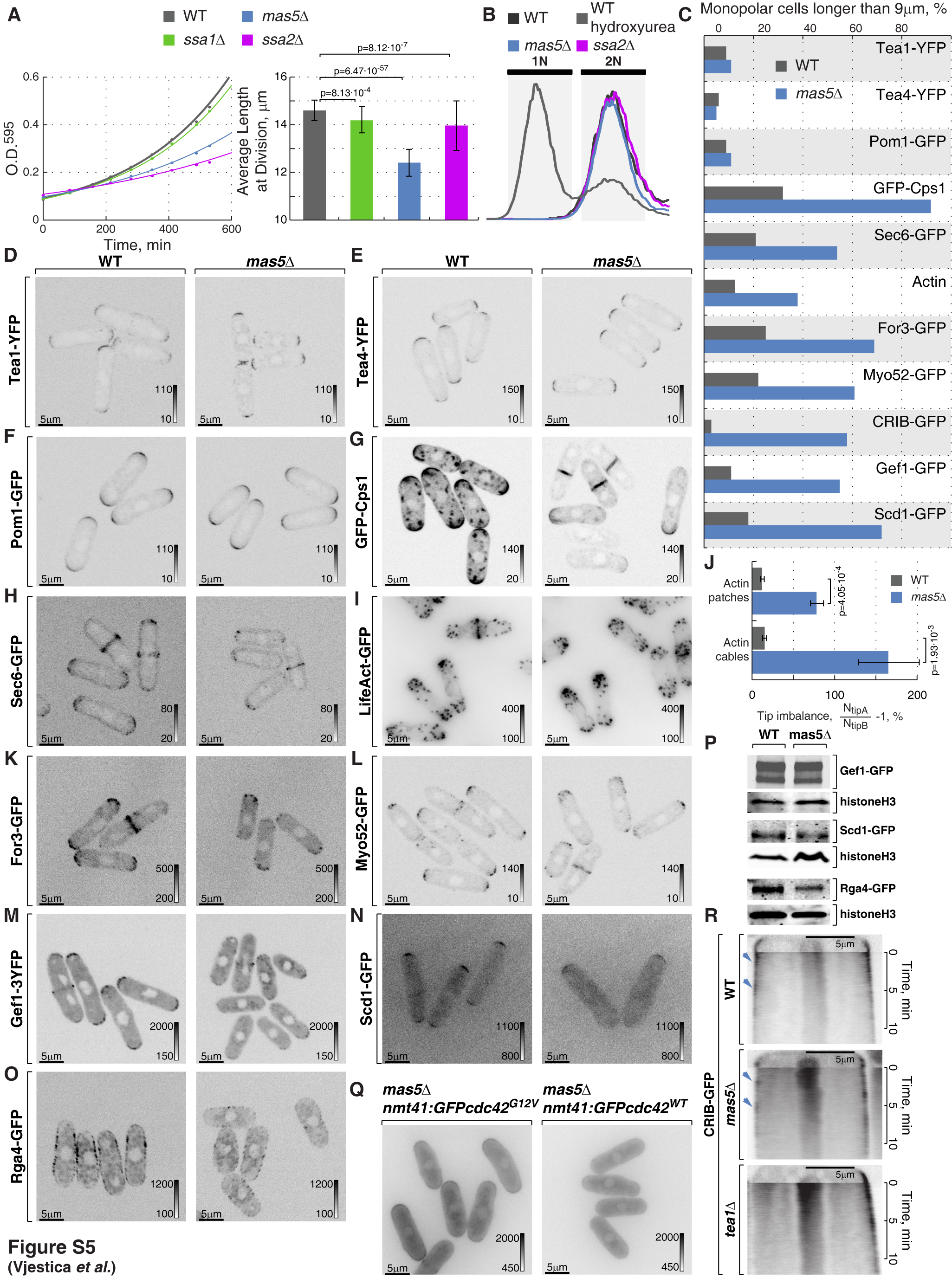

Supplement: Figure S5 — Cells lacking Mas5 or Ssa2 exhibit monopolar intermittent growth in G2 phase of the cell cycle. (A) OD595 nm measurements of log-phase cell cultures of cells with indicated genotypes grown at 24°C (left panel) and average length at division at OD595 nm = 0.5 (right panel). (B) FACS analysis of DNA content for log-phase wild type cells (black), wild type cells arrested in S phase using hydroxyurea (gray) and log-phase ssa2Δ (purple) and mas5Δ cells (blue). (C) Quantification of late G2 cells with monopolar distribution of indicated marker proteins in wild type (gray) and mas5Δ cells (blue). (D–I, K–L) Shown are whole cell maximum intensity z-projections of confocal micrographs of log-phase wild type (left panels) and mas5Δ (right panels) cells expressing indicated fluorophore-tagged marker proteins grown at 24°C. (J) Quantification of the imbalance in number of individual actin structures between localizing to both cell tips in wild type (in gray) and mas5Δ (in blue) late G2 cells grown at 24°C. n>20 per sample. (M–O) Shown are single z-plane micrographs of log-phase wild type (left panels) and mas5Δ (right panels) expressing indicated fluorophore-tagged marker proteins grown at 24°C (left panels). (Q) Single z-plane images of cells with indicated genotypes grown in minimal media in the absence of thiamine for 30 hours and shifted to complete media for 7 hours prior to imaging. Note that the dominant-active mutant Cdc42G12V, but not the wild type protein, is enriched at the cellular cortex. (P) Wild type and mas5Δ cells expressing indicated marker proteins were grown overnight to log-phase (OD595 nm≈0.4) at indicated temperatures prior to total protein extraction. SDS-PAGE resolved protein samples were subjected to Western Blotting using anti-GFP antibodies (top panels). HistoneH3 probing (bottom panels) served as a sample loading control. (R) Kymographs of single z-plane spinning disk confocal microscopy time-lapse analyses of wild type (top panel), mas5Δ (middle pa [file pgen.1003886.s005.tif]

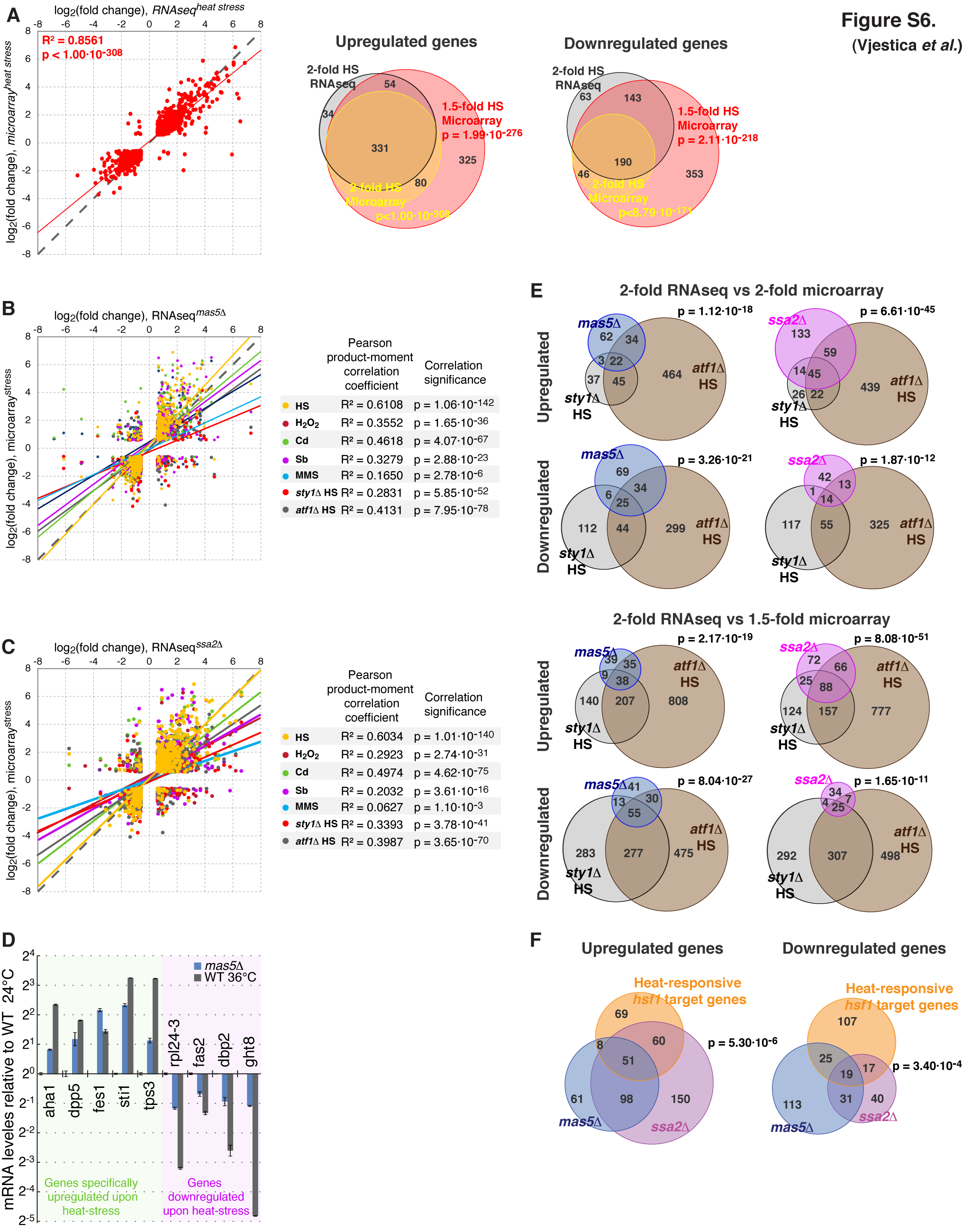

Supplement: Figure S6 — Cells lacking the Ssa2-Mas5 chaperone complex exhibit elevated levels of heat-stress associated transcription. (A) Right panel represents correlation analysis between gene expression profiles of wild type heat-stressed cells analyzed by RNAseq and microarray technologies. Individual dots represent individual genes. Pearson's correlation coefficients and statistical significance are indicated. Venn diagrams analyze the overlap in genes differentially regulated >2-fold (in red), >1.5-fold (in yellow) obtained via microarray (Chen et al., 2003) and >2-fold according to RNAseq (in gray). Correlation analysis between gene expression profiles of mas5Δ (B) or ssa2Δ (C) cells and wild type cells under indicated environmental stresses. Individual dots represent individual genes. (D) Expression levels of genes specifically induced and genes repressed during heat-stress in wild type cells grown at 24°C, shifted to 36°C for 45 min and mas5Δ cells as measured by qPCR. Expression levels are normalized to wild type cells grown at 24°C. (E) Venn diagrams analyze the overlap in genes differentially regulated >2-fold in mas5Δ or ssa2Δ cells and in heat-stressed sty1Δ or atf1Δ cells (Chen et al., 2003). The top set of panels is based on a >2-fold cut-off for data on sty1Δ or atf1Δ cells. The top set of panels is based on a >1.5-fold cut-off for data on sty1Δ or atf1Δ cells. (F) Venn diagrams analyze the overlaps between the gene sets that are differentially regulated >2-fold in mas5Δ or ssa2Δ cells and the heat stress-responsive Hsf1-dependent genes. (TIF) [file pgen.1003886.s006.tif]

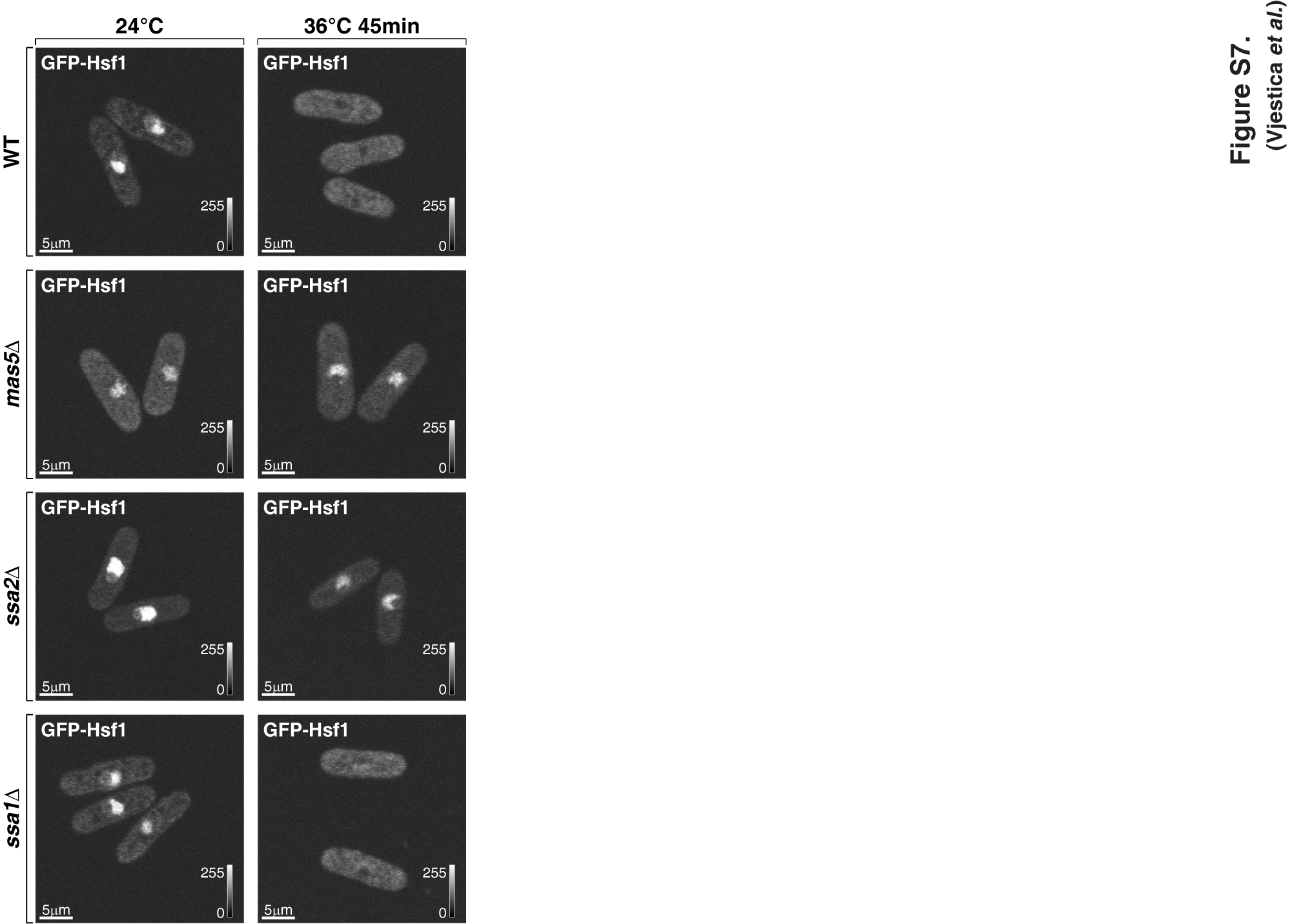

Supplement: Figure S7 — Cells lacking the Ssa2-Mas5 chaperone complex exhibit impaired nucleocytoplasmic shuttling of Hsf1. Shown are whole cell maximum intensity z-projections of scanning confocal micrographs of log-phase cells with indicated genotypes expressing GFP-Hsf1 from its native promoter grown at 24°C (left panels) or shifted to 36°C for 45 min (right panels). Image contrast is reported using corresponding color wedges. Scale bars, 5 µm. (TIF) [file pgen.1003886.s007.tif]
