# Supplementary material for: Hsp70-Hsp40 Chaperone Complex Functions in Controlling Polarized Growth by Repressing Hsf1-Driven Heat Stress-Associated Transcription
Source: PLoS Genet. 2013 Oct 17;9(10):e1003886. doi: 10.1371/journal.pgen.1003886 (PMC3798271; doi:10.1371/journal.pgen.1003886)
Supplement: Table S1 — List of Schizosaccharomyces pombe strains used in this study. (DOCX) [file pgen.1003886.s009.docx]

**Table S1. List of strains used in the study**

| Strain | Genotype | Source |
| --- | --- | --- |
| MBY6656 | *pAct1*:*Lifeact-GFP::leu+* *leu1-32* *ura4-D18* h- | Mohan Balasubramanian |
| SO146 | *tea1-YFP*::kanR *ura4-D18 leu1-32 ade6-M210* h- | Paul Nurse |
| SO1833 | *tea1∆::ura4+* *ura4-D18 leu1? ade?* h- | Paul Nurse |
| SO2865 | *ade6-210 ura4-D18 leu1-32* h+ (MBY102) | Mohan Balasubramanian |
| SO2866 | *ade6-216 ura4-D18 leu1-32* h- (MBY103) | Mohan Balasubramanian |
| SO3483 | *Ssa1-3HA::ura4+* *ade6-210 ura4-D18 leu1-32* h+ | This study |
| SO4281 | *tea4-GFP::kanMX ade6-M21x leu1-32 ura4-D18* h+ | Fred Chang |
| SO4362 | *5’UTR^hsp104^-GFP-3’UTR ^hsp104^::ura4+* @Hsp104 locus *ade6-210 ura4-D18 leu1-32* h+ | This study |
| SO4389 | *mas5∆::ura4+* *ade6-210 ura4-D18 leu1-32* h+ | This study |
| SO4400 | *hsp104-GFP::ura4+* *ade6-210 ura4-D18 leu1-32* h+ | This study |
| SO4594 | *pom1-GFP::*KanMX+ h- | Jürg Bähler |
| SO5216 | *mas5∆::ura4+ sec6-GFP::ura4+leu1-32 ade? ura4-D18* h? | This study |
| SO5217 | *mas5∆::ura4+ tea1-YFP::*kanR *ura4-D18 leu1-32 ade6?* h? | This study |
| SO5274 | *mas5∆::ura4+ pom1-GFP*::KanMX *ura4-D18 leu1-32 ade6?* h? | This study |
| SO5372 | *myo52-GFP::kanR ade6-210 leu1-32 ura4-D18* h- | Dan McCollum |
| SO5374 | *scd1-GFP::kanR leu-? ura-?* h+ | Pilar Perez |
| SO5376 | *pShk1:CRIB^S.cerevisiaeGic2^-3GFP:ura4+ ura4-294 leu1-32* h- (CA5931 rom) | Kazuhiro Shiozaki |
| SO5440 | *mas5∆::ura4+ myo52-GFP::kanR ade6? leu1-32 ura4-D18* h? | This study |
| SO5442 | *mas5∆::ura4+ scd1-GFP::kanR leu-?, ura-?* | This study |
| SO5445 | *mas5∆::ura4+* *pShk1:CRIB^S.cerevisiaeGic2^-3GFP:ura4+ ade6? ura4-? leu1-32* h? | This study |
| SO5448 | *mas5∆::ura4+ lifeact-GFP::leu1+* h? | This study |
| SO5451 | *gef1-3YFP::*kanMX *ade6-704 leu1-32 ura4D-18* h+ | Fulvia Verde |
| SO5452 | *rga4-GFP::*kanMX *ade6-704 leu1-32 ura4D-18* h+ | Fulvia Verde |
| SO5522 | *mas5∆::ura4+ hsp104-GFP::ura4+* *ade6? ura4-D18 leu1-32* h? | This study |
| SO5524 | *mas5∆::ura4+ rga4-GFP::*kanMX *ade6-210 ura4-D18 leu1-32* h? | This study |
| SO5527 | *mas5∆::ura4+* *5’UTR^hsp104^-GFP-3’UTR ^hsp104^::ura4+* @Hsp104 locus *ade6-M21x leu1-32 ura4-D18* h+ | This study |
| SO5666 | *13myc-mas5::ura4+ ade6-210 ura4-D18 leu1-32 h+* | This study |
| SO5672 | *GFP-hsf1::ura4+* *ade6-21x ura4-D18 leu1-32* h- | This study |
| SO5673 | *GFP-hsf1::ura4+ mas5∆::ura4+ ade6-21x ura4-D18 leu1-32* h? | This study |
| SO5683 | *mas5∆::ura4+* *gef1-3YFP::*kanMX *ade6-? leu1-32 ura4D-18* h? | This study |
| SO5778 | *13myc-mas5::ura4+* *ade6-21x ura4-D18 leu1-32* h? | This study |
| SO5873 | *ssa1∆::kanR ade6-216 ura4-D18 leu1-32* h- | This study |
| SO5875 | kanR::*nmt1-hsf1* *ade6-216 ura4-D18 leu1-32* h- | This study |
| SO5946 | kanR*::nmt1-hsf1 Hsp104-GFP::ura4*+ *ade6-21x ura4-D18 leu1-32* h? | This study |
| SO5947 | kanR*::nmt1:hsf1 5’UTR^Hsp104^-GFP-3’UTR^Hsp104^::ura4+* h? | This study |
| SO6017 | *ura4+::nmt81-hsf1* h? | This study |
| SO6393 | *Sec6-GFP::ura4+ ura4-? leu1-32* h- | Mohan Balasubramanian |
| SO6454 | *pNmt3:GFP-cps1*::kanR *ade6-21x ura4?leu1?* h? | Mohan Balasubramanian |
| SO6455 | *pNmt3:GFP-cps1*::kanR *mas5∆::ura4+* *ade6?ura4?leu1?* h? | This study |
| SO6550 | *mas5∆::ura4+* *for3-YFP::*kanR *ade6? ura4-D18 leu1-32* h+ | This study |
| SO6551 | *for3-YFP::*kanR *ade6? ura4-D18 leu1-32* h+ | This study |
| SO6965 | *ssa2∆::ura4+ ade6-21x ura4-D18 leu1-32* h+ | This study |
| SO7020 | *ssa2∆::ura4+ hsp104-GFP::ura4+* *ade6-21x ura4-D18 leu1-32* h? | This study |
| SO7021 | *ssa2∆::ura4+* *5’UTR^hsp104^-GFP-3’UTR ^hsp104^::ura4+* @Hsp104 locus *ade6-21x ura4-D18 leu1-32* h? | This study |
| SO7022 | *ssa2∆::ura4+* *pShk1:CRIB^S.cerevisiaeGic2^-3GFP:ura4+ ade6? ura4-? leu1-32* h? | This study |
| SO7023 | *ssa2∆::ura4+ GFP-hsf1::ura4+ ade6-21x ura4-D18 leu1-32* h? | This study |
| SO7051 | *GFP-ssa2::ura4+* *ade6-21x ura4-D18 leu1-32* h+ | This study |
| SO7052 | *ssa1∆::*kanR *hsp104-GFP::ura4+* *ade6-21x ura4-D18 leu1-32* h? | This study |
| SO7053 | *ssa1∆::*kanR *5’UTR^hsp104^-GFP-3’UTR ^hsp104^::ura4+* @Hsp104 locus *ade6-21x ura4-D18 leu1-32* h? | This study |
| SO7055 | *ssa1∆::*kanR *GFP-hsf1::ura4+ ade6-21x ura4-D18 leu1-32* h? | This study |
| SO7118 | *13myc-mas5::ura4+ GFP-ssa2::ura4+* *ade6-21x ura4-D18 leu1-32* h? | This study |
| SO7701 | *mas5∆::ura4+ p^urg1^:hsf1::*kanR h? |  |
| SO7743 | *mas5∆::ura4+ p^nmt41^:GFP- cdc42G12V::leu1@leu1* |  |
| SO7744 | *mas5∆::ura4+ pnmt41:GFP- cdc42WT::leu1@leu1* |  |
| SO7773 | *p^urg1^:hsf1::*kanR *hsp104-GFP::ura4+* |  |
| SO7777 | *mas5∆::ura4+ p^urg^1:hsf1::*kanR *hsp104-GFP::ura4+* |  |
