# Supplementary material for: Hsp70-Hsp40 Chaperone Complex Functions in Controlling Polarized Growth by Repressing Hsf1-Driven Heat Stress-Associated Transcription
Source: PLoS Genet. 2013 Oct 17;9(10):e1003886. doi: 10.1371/journal.pgen.1003886 (PMC3798271; doi:10.1371/journal.pgen.1003886)
Supplement: Table S2 — List of strains used in the screening for cells with elevated levels of heat stress-associated transcription. (DOCX) [file pgen.1003886.s010.docx]

**Table S2. List of strains used in the screening for cells with elevated heat-stress transcription (Fig. 4)**

| **Viability  (Kim *et al.*, 2010)** | **Cellular subcompartment (Matsuyama *et al.*, 2006)** | **Systematic Id** | **Product** | **Description** |  |
| --- | --- | --- | --- | --- | --- |
| Inviable | Endoplasmic reticulum | SPBC1347.05c |  | DNAJ domain protein Scj1 (predicted) | **Absent from  the screening library** |
| Inviable | Endoplasmic reticulum Golgi | SPAC17A5.12 | ucp7 | UBA/TPR/DNAJ domain protein Ucp7 |  |
| Inviable | Mitochondrial | SPCC4G3.14 | mdj1 | mitochondrial DNAJ domain protein Mdj1 (predicted) |  |
| Not determined | Mitochondrial | SPAC824.06 | tim14 | TIM23 translocase complex subunit Tim14 (predicted) |  |
| Viable | Endoplasmic reticulum | SPBC36B7.03 | sec63 | ER protein translocation subcomplex subunit Sec63 (predicted) |  |
| Viable | Mitochondrial | SPAC144.08 |  | mitochondrial DNAJ domain protein Jac1 (predicted) |  |
| Viable | Mitochondrial | SPAC24H6.02c |  | TIM23 translocase complex subunit Tim15 (predicted) |  |
| Viable | Nucleo-cytoplasmic | SPBC1773.09c | mug184 | meiotically upregulated gene Mug184 |  |
| Viable | Nucleo-cytoplasmic | SPBC1734.05c | spf31 | DNAJ protein Spf31 (predicted) |  |
| Viable | Nucleo-cytoplasmic | SPAC1071.09c |  | DNAJ domain protein, DNAJC9 family (predicted) | **Present in  the screening library** |
| Viable | Nucleo-cytoplasmic | SPAC2E1P5.03 |  | DNAJ domain protein Erj5 (predicted) |  |
| Viable | Nucleo-cytoplasmic | SPAC4G9.19 |  | DNAJ domain protein DNAJB family (predicted) |  |
| Viable | Nucleo-cytoplasmic | SPAC4H3.01 |  | DNAJ domain protein Caj1/Djp1 type (predicted) |  |
| Viable | Nucleo-cytoplasmic | SPAC6B12.08 | mug185 | Co-chaperone for ATPase activity (predicted) |  |
| Viable | Nucleo-cytoplasmic | SPAC926.05c | dph4 | diphthamide biosynthesis protein Dph4 (predicted) |  |
| Viable | Nucleo-cytoplasmic | SPBC11B10.05c | rsp1 | random septum position protein Rsp1 |  |
| Viable | Nucleo-cytoplasmic | SPBC1734.11 | mas5 | DNAJ domain protein Mas5 (predicted) |  |
| Viable | Nucleo-cytoplasmic | SPBC1778.01c | zuo1 | zuotin (predicted) |  |
| Viable | Nucleo-cytoplasmic | SPBC17A3.05c |  | DNAJ/DUF1977 DNAJB12 homolog (predicted) |  |
| Viable | Nucleo-cytoplasmic | SPBC3E7.11c |  | DNAJ protein Caj1/Djp1-type (predicted) |  |
| Viable | Nucleo-cytoplasmic | SPBC405.06 |  | DNAJ protein Xdj1 (predicted) |  |
| Viable | Nucleo-cytoplasmic | SPBC543.02c |  | DNAJ/TPR domain protein DNAJC7 family |  |
| Viable | Nucleo-cytoplasmic | SPCC10H11.02 | cwf23 | DNAJ domain protein Cwf23 |  |
| Viable | Nucleo-cytoplasmic | SPCC63.03 |  | DNAJ domain protein, DNAJC11 family |  |
| Viable | Nucleo-cytoplasmic | SPCC63.13 |  | DNAJ domain protein |  |
| Viable | Nucleo-cytoplasmic | SPCC830.07c | psi1 | DNAJ domain protein, involved in translation initiation Psi1 |  |
