# Supplementary material for: Hsp70-Hsp40 Chaperone Complex Functions in Controlling Polarized Growth by Repressing Hsf1-Driven Heat Stress-Associated Transcription
Source: PLoS Genet. 2013 Oct 17;9(10):e1003886. doi: 10.1371/journal.pgen.1003886 (PMC3798271; doi:10.1371/journal.pgen.1003886)
Supplement: Table S3 — List of primers used in the qPCR analysis of differential gene expression in heat-stressed wild type and mas5Δ cells. (DOCX) [file pgen.1003886.s011.docx]

**Table S3. List of primers used in the qPCR analysis**

| prSO2601 | ATTACTTGCCCCGAGCTTTC | forward qPCR primer for SPBC1711.08 (*aha1*) |
| --- | --- | --- |
| prSO2602 | ACTATGCACGGGAAGTCCAC | reverse qPCR primer for SPBC1711.08 (*aha1*) |
| prSO2603 | TGGATTGTAAAGCCCGAAAG | forward qPCR primer for SPBC1711.12 (*dpp5*) |
| prSO2604 | GCTTACCACCCCAATCAAGA | reverse qPCR primer for SPBC1711.12 (*dpp5*) |
| prSO2605 | AGTACGCAGTTGCAAGCTCA | forward qPCR primer for SPBC3B9.01 (*fes1*) |
| prSO2606 | CCGGACTCTCAAGCTGTTTC | reverse qPCR primer for SPBC3B9.01 (*fes1*) |
| prSO2607 | GCCGACTTTAAGCTCATTGC | forward qPCR primer for SPCC645.14c (*sti1*) |
| prSO2608 | GGCTTTGTCAGGGTCAATGT | reverse qPCR primer for SPCC645.14c (*sti1*) |
| prSO2609 | GCTGCTTCTTGGGCAGTTAC | forward qPCR primer for SPACUNK4.16c (*tps3*) |
| prSO2610 | GCGCACTGAAAACGTAAACA | reverse qPCR primer for SPACUNK4.16c (*tps3*) |
| prSO2611 | ACCGTAAGGCACATGGAAAG | forward qPCR primer for SPAC22E12.13c (*rpl24*-*3*) |
| prSO2612 | GGGGTTCTTGTACCTCAGCA | reverse qPCR primer for SPAC22E12.13c (*rpl24*-*3*) |
| prSO2613 | CTCAAAAATGCCTGGTGGTT | forward qPCR primer for SPAC4A8.11c (*fas2*) |
| prSO2614 | AAGAACCGCCTTCAGCAGTA | reverse qPCR primer for SPAC4A8.11c (fas2) |
| prSO2615 | ATTCGACCAGACCGTCAAAC | forward qPCR primer for SPBP8B7.16c (dbp2) |
| prSO2616 | CCAACCATCTTGCCTAAGGA | reverse qPCR primer for SPBP8B7.16c (dbp2) |
| prSO2617 | TTCGCTGCCTGTAATTTGTG | forward qPCR primer for SPCC548.06c (ght8) |
| prSO2618 | CACGGCCTTCAGAAGAAGAG | reverse qPCR primer for SPCC548.06c (ght8) |
| prSO2621 | AACCCTCAGCTTTGGGTCTT | forward qPCR primer for SPBC32H8.12c (act1) |
| prSO2622 | ATTTACGCTCAGGAGGAGCA | reverse qPCR primer for SPBC32H8.12c (act1) |
| prSO2623 | CCGATGTTTCCGTTGTTGAC | forward qPCR primer for SPBC354.12 and SPBC32F12.11 (gpd1) |
| prSO2624 | ACGAGCTTGACGAATTGAGG | reverse qPCR primer for SPBC354.12 and SPBC32F12.11 (gpd1 ) |
